# Shaping bacterial population behavior through computer-interfaced control of individual cells

**DOI:** 10.1038/s41467-017-01683-1

**Published:** 2017-11-16

**Authors:** Remy Chait, Jakob Ruess, Tobias Bergmiller, Gašper Tkačik, Călin C. Guet

**Affiliations:** 1 Institute of Science and Technology Austria, Klosterneuburg, 3400 Austria; 2 Inria Saclay, Ile-de-France, Palaiseau 91120 France; 3 Institut Pasteur, 75724 Paris Cedex 15, France

## Abstract

Bacteria in groups vary individually, and interact with other bacteria and the environment to produce population-level patterns of gene expression. Investigating such behavior in detail requires measuring and controlling populations at the single-cell level alongside precisely specified interactions and environmental characteristics. Here we present an automated, programmable platform that combines image-based gene expression and growth measurements with on-line optogenetic expression control for hundreds of individual *Escherichia coli* cells over days, in a dynamically adjustable environment. This integrated platform broadly enables experiments that bridge individual and population behaviors. We demonstrate: (i) population structuring by independent closed-loop control of gene expression in many individual cells, (ii) cell–cell variation control during antibiotic perturbation, (iii) hybrid bio-digital circuits in single cells, and freely specifiable digital communication between individual bacteria. These examples showcase the potential for real-time integration of theoretical models with measurement and control of many individual cells to investigate and engineer microbial population behavior.

## Abstract (web summary)

Individual bacteria interact with each other and their environment to produce population-level patterns of gene expression. Here the authors use an automated platform combined with optogenetic feedback to manipulate population behaviors through dynamic control of individual cells.

## Introduction

Predicting the behavior of individual bacteria and bacterial populations is challenging and the complexity of the task increases rapidly already in the simplest laboratory conditions that include population heterogeneity and ecological or environmental interactions^1^. Even clonal groups of microbes can interact with each other and with nearby organisms^1–6^, undergo spatial and functional organization^1,6–9^, insulate their populations from transient stresses, including antibiotics^6,10^, and coordinate virulence^11–13^. Therefore, to understand and manipulate natural or engineered bacterial populations, we require the ability to experimentally measure and control factors in individual cells that generate emergent population behaviors.

Recent technological advances have facilitated experiments at the single-cell level in defined conditions. Microfluidic devices enable long-term observation of individual cells and precise environmental control^14–16^. However, differentially perturbing many individual cells is technically involved. Molecular genetics techniques permit straightforward design of synthetic genetic circuits to assay their effects at the population level^17,18^. However, in vivo behavior of even simple synthetic circuits is often hard to predict, and disentangling interactions between their components and with the host remains a laborious task^19–22^. Finally, computer-interfaced chemical and optogenetic methods of gene regulation offer new tools for specified modulation of microbial gene expression^23–30^. As yet, these methods have either been applied uniformly across populations, or in certain cases to a single cell. Online measurement and gene expression control in many individual cells at once is still lacking. Such a capability would provide a powerful way to probe and control microbial populations, including collective behaviors of populations that originate at the single-cell level.

To this end, we constructed a general purpose, automated platform to programmatically measure and control gene expression in lots of individual bacterial cells over many generations, while dynamically modulating the chemical environment of the cells. The platform we developed combines microfluidics and optogenetics and enables simultaneous, quantifiable light-responsive control of gene expression over several days in hundreds of individual bacteria, as well as global chemical perturbation (e.g. nutrient shifts, toxin exposure). The platform is run by a computer that defines and controls the entire experiment, analyzes the data online, and uses independent software controllers to automatically adjust scheduled light perturbation sequences on the fly for each individual bacterium. In the following, we introduce the platform and show how it provides straightforward access to important general characteristics of microbial populations.

## Results

### Experimental setup

**Fig. 1:**
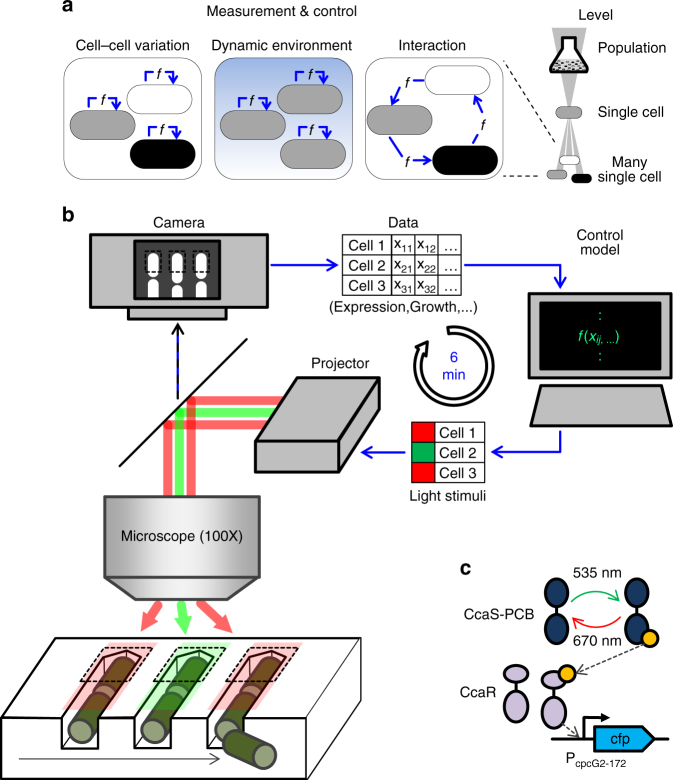
An experimental platform for independently programmable optogenetic control of gene expression in individual bacteria. **a** Online measurement and control (cell out-, in-arrows) coupled through an experimental platform (*f*) of many single bacteria enables probing single-cell heterogeneity, effects of environmental variability in individual cells, and group behaviors of interacting individuals. **b** Experimental platform overview: individual bacteria are cultured for days in defined chemical environments within a microfluidic “mother machine” device. Fluorescent reporter expression, cell shape, and growth rate of the mother cell at each control location (dashed box) is automatically extracted from microscope images and provided to the cell’s individually specified software controller. The controllers output stimuli to up- or downregulate a light-responsive gene for each cell. The individual stimuli are collected, spatially arranged and transmitted to the recipient cells using a custom-modified microscope-coupled LCD projector. This process is repeated every 6 min. **c** Cerulean CFP expressed via an optimized CcaSR optogenetic regulation system^31^. CcaS-phycocyanobilin autophosphorylates under green light (535 nm), then phosphorylates CcaR, which binds and activates expression from the PcpcG2-172 promoter. Exposure to red light (670 nm) dephosphorylates CcaS, eventually halting expression

We constructed the setup outlined above to perform a measurement-and-control loop (Fig. 1a, b, Methods) on *E.coli* cells bearing a light-regulated gene transcription module. Long-term control of individual cells necessitates a microculture environment that can operate stably for hundreds of generations. We therefore employ a microfluidic “mother machine” device to grow and track the individual cells confined at closed ends of short (~23 μm) cell-width channels over hours or days on a temperature-controlled fluorescence microscope (Methods, Supplementary Figs. 1 and 2)^14^. In these devices, larger channels intersect the growth channels, supplying fresh nutrient media and chemical perturbations and removing waste and progeny of each channel’s focal “mother” cell. Gene expression is estimated using image intensities of a fluorescent reporter. Since reporter levels vary too much for reliable segmentation, morphological cell data are acquired by imaging a second, constitutively expressed, fluorescent reporter. Software controllers, associated with individual cells or cell groups, process these data and return expression activation/repression signals for delivery to each cell. Cells are individually stimulated by projecting an RGB image of the signal intensities, mapped to appropriate color channel and cell locations, onto the light-responsive cells using a modified overhead projector (Methods, Supplementary Figs. 1 and 2, Supplementary Movie 1). Maximum cell numbers are thus constrained by the time required to visit and set up each field of view, perform data acquisition, analysis and control computations, and stimulate every cell at a sufficient frequency. In our experiments, six minute control intervals permit initial tracking and control of 200–400 cells in four to eight fields of view at ×100 magnification, with maximum cell attrition-limited durations of several days (Methods).

In closed-loop mode, once defined, experiments are implemented entirely programmatically and without intervention. To enable fully autonomous operation, we wrote custom MATLAB software to manage all aspects of the experiment (Methods). The modular software eases modifications of the physical system, and simplifies revision of controllers and experiment protocols.

To independently regulate expression in single cells, we rely on light-responsive transcription mechanisms. We employ a recently optimized *ccaSR*-based system with low leak and large dynamic range, which includes a synthesis pathway for the necessary phycocyanobilin (PCB) chromophore^31^. Illumination with green (~535 nm) or red (~670 nm) light, respectively, activates or deactivates expression from the P_cpcG2-172_ promoter with timescales on the order of several minutes, without differentially affecting growth^27,31,32^. For testing our setup, we have placed a cyan fluorescent protein expression reporter, Cerulean (cfp), under the control of the P_cpcG2-172_ promoter (Fig. 1c).

### Single-cell control

We verified our system by controlling gene expression patterns in a small population of *Escherichia coli*. In cell population control, mean and individual error represent two important deviations of expression from target levels, and depend broadly on the control type (Table 1).

**Table 1:** Error level reduction by control type

Control type	Mean error reduction	Individual error reduction
Open loop (OL)	No	No
Population-level closed loop (pCL)	Yes	No
Individual-level closed loop (iCL)	Yes	Yes

Open loop (OL) controllers precompute light stimulation sequences based on an average cell response model. OL controllers suffer from both mean and individual error. Mismatches between modeled and actual population responses produce systematic errors in mean expression that are sensitive to unmodeled changes in cell behavior (e.g., with environment). Further, “average cell” stimuli fail to account for cell-to-cell differences in response, and cannot contain the resulting variation in expression. By comparing expected and measured responses to the control stimulus, population-level closed loop (pCL) controllers substantially reduce mean target deviations by adjusting global stimuli on-the-fly^23,24,27,33^. However, stimuli applied uniformly across the population cannot contain variation stemming from differences between cells. To reduce such individual error, controllers should operate at the single cell level at which the error is generated. Closed loop control has been demonstrated at the level of a single yeast cell^26^, and we tested whether extending this to parallel, individual-level closed loop (iCL) control of a population of many cells within our device could mitigate both sources of error.

**Fig. 2:**
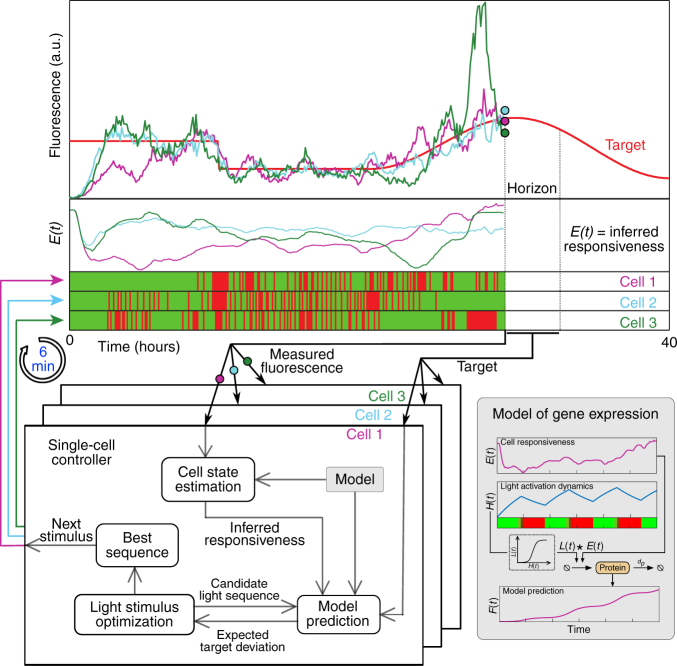
Parallelized model predictive control of individual cells. Single cell controllers iteratively use measured fluorescence trajectories (examples for three individual cells, top) and a Kalman filter to infer cells’ transcriptional responsiveness, *E*(*t*), based on past activating and deactivating light sequences (green, red series), and suggest the next stimuli from light sequences that minimize the error between expected future fluorescence levels and a target profile (red line) within a specified planning horizon. To calculate expected fluorescence levels and infer responsiveness, the controllers make use of a simple stochastic model of gene expression (gray box, bottom right, see Supplementary Methods) consisting of three state variables that represent light activation *H*(*t*), cell responsiveness *E*(*t*), and fluorescence *F*(*t*). The same control scheme could use different, potentially more complex, models of gene expression, by simply swapping the content of the “Model” module. See Supplementary Methods for detailed discussion of models and controllers used in this study, and their interaction with cells

To control gene expression in individual cells, we used a receding-horizon control scheme (Fig. 2) based on a simplistic (although predictive) stochastic kinetic model that we identified from several calibration experiments (Supplementary Methods). The model incorporates an internal (unmeasured) state, hereafter termed “cell responsiveness” (Fig. 2) that can vary between cells and in time. Every 6 min, for each cell, the controller compares the recorded fluorescence level to a predicted level calculated from the model and updates its estimate about the cell’s responsiveness by weighting prediction and measurement according to their uncertainties. Measurement uncertainty stems from technical errors in recording cells’ fluorescence whereas prediction uncertainty is a consequence of stochasticity in modeled chemical reactions and the imperfectly known, possibly time-varying, cell responsiveness. The prediction uncertainty can be efficiently calculated from the stochastic model of the system using moment equations (Supplementary Methods). The controller then uses the updated estimate of the cell’s responsiveness to identify a light sequence that minimizes the deviation of the expected fluorescence levels in the cell from the desired target profile over a certain planning horizon (Fig. 2).

**Fig. 3:**
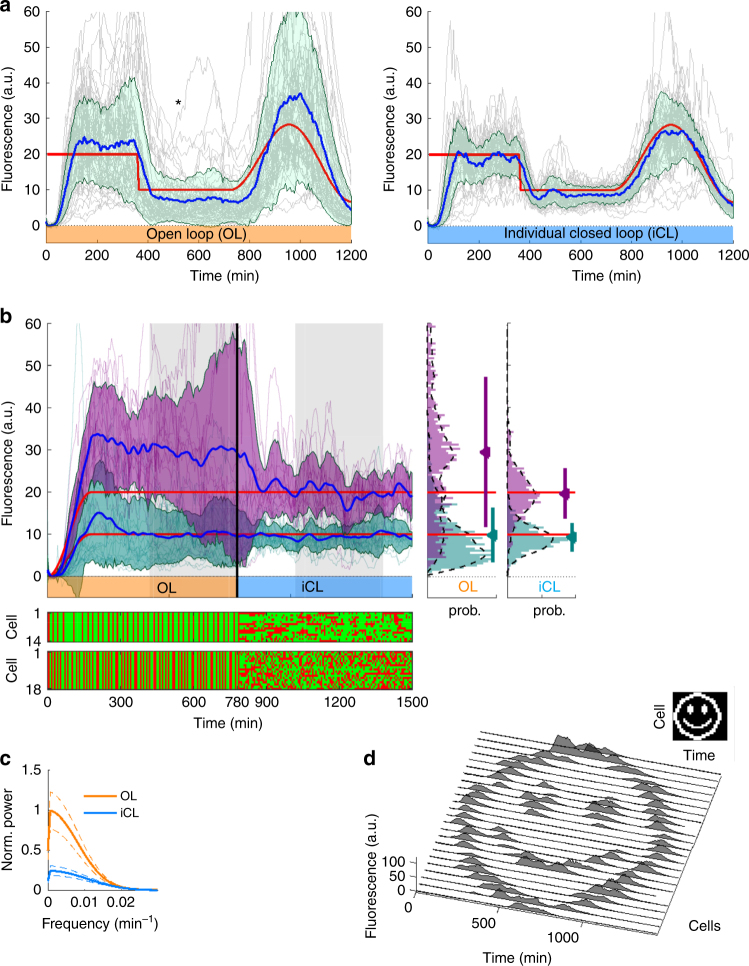
Specifying gene expression distributions in small bacterial populations using iCL control. **a** CFP intensity trajectories (gray lines) for individual *Escherichia coli* cells tracking targets (red lines) under model-based control in either OL (left panel, *n* = 55 cells) or iCL mode (right panel, *n* = 49 cells), pooled from two replicate experiments. Deviations of average population trajectories (blue lines) from targets represent errors in control of the mean. Expression distribution breadths (shaded regions, ±1 s.d.) indicate errors in controlling individual cells. iCL control reduces mean error and cell-to-cell variation, and removes extended excursions in responsiveness seen with OL control (e.g., asterisk-labeled CFP trajectory, Supplementary Movie 3). **b** Individual CFP trajectories (purple, teal lines) and mean trajectories ±1 s.d. (blue lines and purple, teal-shaded regions, 1 h moving average) for subsets of cells controlled with targets (red lines) of 10 a.u. or 20 a.u. (*n* = 18, 14 cells, respectively). All cells switch from OL (orange-labeled time interval) to iCL (blue interval) control at 780 min. Individual light stimulation sequences (green: activation, red: repression) for cells targeted to 20 a.u. (upper sequences) and 10 a.u. (lower sequences) are displayed below the plot. Distributions of CFP levels in OL and iCL regimes (during gray shaded intervals) are shown to the right. iCL control (right plot), resolves cells into target group distributions (purple, teal distributions, smoothed: dashed lines) by reducing large errors between target group means (triangles) and expression targets (red lines), and narrowing individual errors (error bars: ±1 s.d.). **c** Average power spectral densities of OL- and iCL-controlled (orange, blue) fluorescence trajectories (in gray shaded intervals) in **b**. iCL control reduces fluctuations at frequencies below 0.02 min^−^^1^. Average power spectral estimates are for 14 mean-subtracted trajectories with constant fluorescence target of 20 a.u. (spectra are similar for 18 cells targeting 10 a.u.). Dashed lines denote standard error of the mean. **d** Single-cell temporal patterning of CFP expression in 24 cells over 24 h. iCL control targets are rasters of a binarized image (inset) with fluorescence levels 0 and 15 a.u. Cells shown are automatically selected by longest-validity (Methods) from eight simultaneous replicates

We tested this control scheme by making individual cells track a target fluorescence profile consisting of steps and a sine-wave. To quantify control performance, we split the cells into two groups. For the first group, we used OL control to pre-calculate the entire optimal light sequence (based on a mean population response model identified from calibration experiments) without online learning of the responsiveness or feedback of the cells. The second group was iCL feedback-controlled as described in Fig. 2 (see sample cells, Supplementary Movie 2). The resulting mean fluorescence of the OL-controlled group of cells (Fig. 3a, left panel, blue line) is roughly correct, implying that our stochastic model is predictive over long-time horizons (see ref. ^25^ for further results of this type). However, the control performance is less satisfactory for individual cells (gray lines). Heterogeneity in a population exposed to an identical light stimulation pattern results in widely varying fluorescence trajectories across the cells and, thus, high variance around the mean response. In particular, fluorescence of some cells is either far above or far below the average for long periods of time. We expect individualized light patterns based on online learning of each cell’s responsiveness to decrease stimulation of over-responsive cells and increase it for under-responsive cells, reducing both the mean error and expression heterogeneity of the population. Indeed, the iCL-controlled cells exhibit both a reduced error in mean fluorescence, and a narrower distribution around that mean than cells under OL control (Fig. 3a, right panel, See Supplementary Methods for a comparison of pCL and iCL controllers).

### Defining population expression distributions

The effectiveness of iCL control in reducing mean error and cell-to-cell variation in expression suggested that —beyond simply narrowing population expression around single targets—we could use such control to specify more complex distributions of gene expression. Recent studies suggest that phenotypic distributions in isogenic populations could have strong fitness effects, e.g., as bet hedging strategies for rare but toxic environments^6,15,34,35^. Testing such ideas rigorously would benefit from an experimental capacity to specify desired expression distributions and expose them to precisely modulated environments.

To evaluate iCL control for specifying expression distributions, we targeted halves of a population to two nearby CFP fluorescence levels (10, 20 a.u). The experiment ran for 780 min in OL mode (precomputed, global light stimulation) to steer expression to the two targets (Fig. 3b). While mean population fluorescence levels initially approach the targets, there are large residual errors in the mean and high variability. Both mean and individual errors interfere with separating the joint population into clearly defined subpopulations with distinct targets (Fig. 3b, center right panel). After 780 min, the controllers switched to iCL mode, adjusting stimulation sequences based on individual cell responses (Fig. 3b, bottom; Supplementary Movie 4). Shortly thereafter, mean error and variation within each subpopulation are sharply reduced. Comparing average mean-subtracted power spectra of individual cells’ expression during OL and iCL control, we observe that iCL control reduces amplitudes of slow expression fluctuations (*f* < 1 h^−1^; Fig. 3c). The population resolves into two accurately targeted groups of cells (Fig. 3b, rightmost panel). Reversing the control order and switching from iCL to OL has the opposite effect (Supplementary Fig. 3).

Independent and parallel control of expression in individual cells enables specifying not only stationary distributions, but also precise temporal gene expression patterns in groups of cells. As an example, we used our setup to straightforwardly program a population of 24 cells under iCL control to track distinct dynamic CFP expression targets over a 24 h interval (Fig. 3d).

### Population variability control under antibiotic perturbation

A key use of our platform is to probe how populations with distributed phenotypes interact with changing environments. Such investigations depend on our ability to modify the environment precisely while maintaining a desired phenotypic distribution. The microfluidic devices we use for long-term culture of individual bacteria are uniquely suited to exert precise chemical and temporal control over cells’ environments by switching between media sources. In our setup, we switch media with 1–10 min lag at junctions upstream of the device.

For a simple test of combined environmental manipulation and expression control, we elaborated our control experiments to include a switch from antibiotic-free media to media containing a sub-inhibitory level of the translation-inhibiting tetracycline antibiotic, doxycycline^36^. Sub-inhibitory antibiotic concentrations can generate diverse behaviors by modifying gene expression and cell physiology^37^. Such changes are difficult to capture in simplified models, and as cells are driven further from the regime in which the control model was identified, the increasing mismatch between model and cell should amplify the mean error between cell fluorescence and control target. Additionally, antibiotics acting on cells with small initial differences in susceptibility could increase variability (and thus mismatch with the average cell) within a population^38^.

**Fig. 4:**
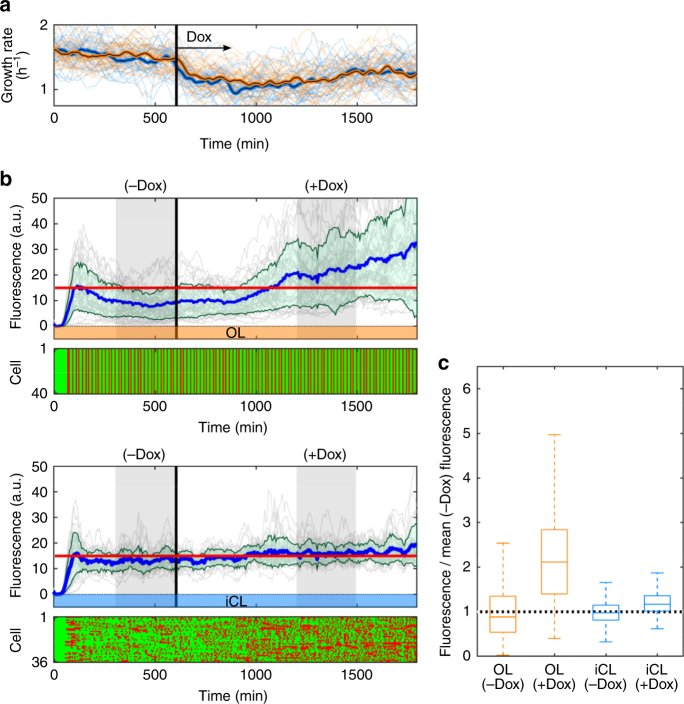
iCL control maintains low mean and individual errors in a population perturbed with antibiotic. **a** Growth rates (individual: light lines; mean: dark lines) of cells under open loop (OL) control (orange, *n* = 40 cells), and individual closed loop (iCL) control (light blue, *n* = 36 cells). Growth slows after 0.6 μg ml^−1^ doxycycline is added to the culture media at 600 min (vertical line). OL and iCL data are acquired simultaneously from equal numbers of cells initialized per field of view to maximize environmental correspondence. **b** Individual and mean CFP fluorescence trajectories (gray, blue lines respectively; green shaded regions = mean±1 s.d.) of cells tracking a constant target (15 a.u.) under OL control (upper panel) or iCL control (lower panel). Light stimulation sequences are displayed below the fluorescence plots. **c** Relative perturbation of pre-antibiotic CFP fluorescence levels (measured in −Dox shaded region in **b**) by doxycycline (measured in +Dox shaded region in **b**), displayed as box plot distributions for open loop (OL, orange) or individual closed loop (iCL, blue) controlled cells. To assess the sensitivity of OL and iCL control to the perturbation, data are normalized, per controller, to mean fluorescence levels within a 5-h interval immediately prior to doxycycline exposure (−Dox, shaded region in **b**), for comparison to a second interval (+Dox, shaded region in **b**) between 10 and 15 h after doxycycline addition

We assessed the effect of doxycycline on population and individual control errors by measuring the change in mean and variance of an OL-controlled population with a single fluorescence target (15 a.u.) upon antibiotic exposure. To test how closed-loop control moderates these effects, we assigned a similar number of cells to the iCL control algorithm. We allowed cells 10 h to acclimatize in the absence of doxycycline before switching to media containing 0.6 μg ml^−1^ of the antibiotic, which rapidly slowed the average growth of both OL and iCL-controlled populations by ~30% (Fig. 4a, Methods). Since at minimum, this gross shift in dilution rate is not accounted for by our simple model of gene expression that assumes a constant growth rate identified in the absence of doxycycline, we expect its predictive accuracy to decline in the presence of the antibiotic. Indeed, following the growth rate shift, the mean fluorescence of the OL-controlled cells diverges above the target level without stabilizing through an additional 1200 min of antibiotic exposure (Fig. 4b and Supplementary Fig. 4). Note that mean fluorescence remains stable for approximately 300 min following the growth shift, suggesting that “fixing” the model would require more than simply incorporating active growth rate measurements. Importantly, because our platform processes cell fluorescence data online, it can detect and respond to effects of changing environmental conditions in real time by appropriately adjusting light inputs. Our simple predictive model of gene expression captured the effects of doxycycline perturbation as an increase in cells’ responsiveness, informing the iCL control algorithm that less activating green light is required to maintain stable fluorescence levels. The mean fluorescence of the iCL-controlled population thus experiences only a slight, stable increase, without an appreciable increase in population variability (Fig. 4b and Supplementary Fig. 4). The small shift in the iCL-controlled mean results from the aforementioned model mismatch under antibiotic-containing conditions relative to the conditions used for model identification (see Supplementary Information). Comparison of the probability distributions of OL and iCL-controlled cells in 300 min intervals prior to and during antibiotic exposure (Fig. 4c and Supplementary Fig. 4) indicates that iCL control largely mitigates both population and individual errors stemming from model-environment mismatch, ultimately with iCL-controlled expression only slightly affected by the antibiotic.

### Bio-digital circuits and specifiable cell–cell interactions

Finally we tested an idea that, besides explicitly controlling gene expression, our platform could digitally virtualize elements of transcriptional circuits. Such bio-digital circuits would permit powerful, facile specification of properties of their digital component (e.g., dynamics, connectivity, response, noisiness), while retaining their in vivo context for assay.

**Fig. 5:**
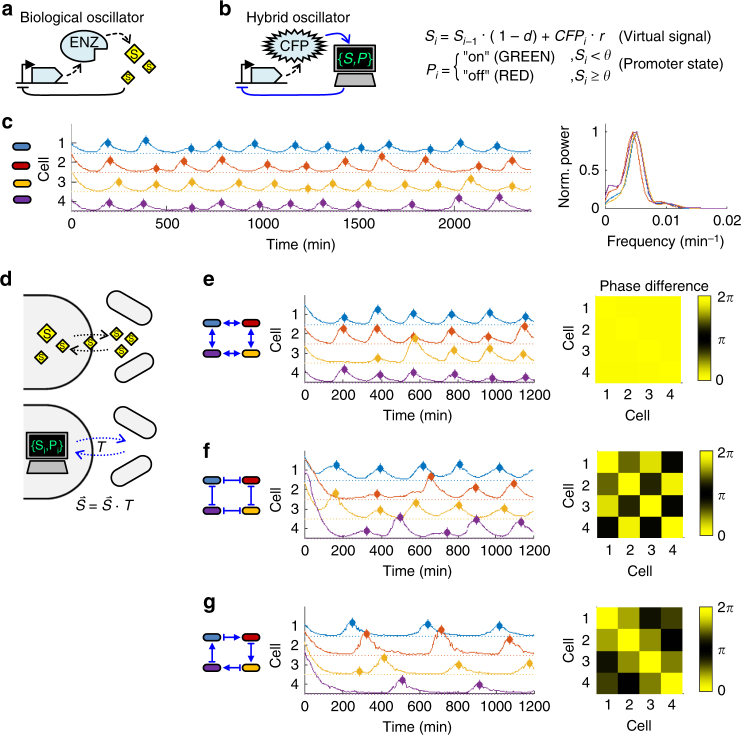
Digitally specified communication between single-cell hybrid oscillators. **a** Simplified scheme for a biological oscillator driven by a delayed negative feedback^39^ on expression of an enzyme (ENZ) by an enzyme-produced inhibitory signal (S). **b** Analogous architecture of a hybrid oscillator with virtualized inhibitory signal (*S*). A CFP expression reporter and light-responsive promoter interface the biological side of the circuit to a digital component that implements a discrete time CFP-dependent accumulation of virtual inhibitory signal (production rate *r*, removal rate *d*) and exposes cells to red or green light stimuli determined by virtual signal-dependent threshold (*θ*) activation of the digital promoter state (*P*). **c** Raw CFP fluorescence trajectories of four single-cell hybrid oscillators over 40 h (left panel; *r* = 3, *d* = 0.2, *θ* = 60). Filled diamonds denote expression peaks of the trajectories (fit after local smoothing), for comparing oscillation timing between cells. Median trough, peak fluorescence: 2.0 a.u., 10.9 a.u., respectively. Power spectra (right panel) of the (mean-subtracted) trajectories exhibit a common peak frequency around 0.005 min^−1^. **d** Biological oscillators can be coupled by transporting a chemical signal, S, across cell boundaries (top). The hybrid oscillators can similarly distribute a virtual signal between cells by multiplying their signal vector, $\mathop{S}\limits^{\rightharpoonup}$, with a digitally specified transfer matrix, *T*, at each time step. **e** CFP trajectories of four coupled hybrid oscillators (left schematic and middle panel; *r* = 3, *d* = 0.2, *θ* = 60, for every cell, 0.1*S* is transferred to the nearest neighbors, per interval). Coincidence of expression peaks (filled diamonds) and near zero phase difference (right panel) indicate synchrony of the oscillators. Median trough, peak fluorescence: 1.8, 13.6 a.u. (**f**, **g**). CFP trajectories (middle panel; filled diamonds: expression peaks) and phase difference (right panel) of 4-member **f** negatively coupled (*r* = 3, *d* = 0.2, *θ* = 60, for every cell, 0.1*S* is removed from the nearest neighbors per interval; median trough, peak fluorescence: 3.2, 18.9 a.u.), and **g** asymmetrically coupled (*r* = 3, *d* = 0.16, *θ* = 60, for every cell, 0.1*S* is respectively added and removed from the left- and right-hand nearest neighbors, per interval; median trough, peak fluorescence: 1.5, 16.3 a.u.) groups of hybrid oscillators

We therefore produced a toy hybrid version of an established circuit, an oscillator, composed of interfaced biological and digital components. Extensively studied natural genetic oscillators exhibit quantifiable phenotypes of frequency, phase, and amplitude^39^. Simple synthetic oscillators have been tested as exploratory tools and sensor readouts^40,41^. A common core architecture among biological oscillators is a delayed negative feedback loop (Fig. 5a), in which a chemical signal (S) generated by a gene product (ENZ) eventually represses the expression of the gene itself^39^. In particular regimes, the inhibitory signal is then cyclically depleted until expression resumes, producing stable oscillations^39^.

We virtualized the inhibitory signal component, digitally specifying a gene product-dependent production rate (*r*), first order removal rate (*d*), and its interaction with the gene’s promoter via a digital promoter state, *P* (Fig. 5b). To measure gene product levels without producing real inhibitory signal, we replaced the gene with a CFP reporter, thus interfacing the biological to the digital side of the network. At each 6 min measurement and control interval (*i* = 1,2,3,…) the virtual signal *S*_*i*_ accumulates in proportion to a cell’s measured CFP fluorescence and is removed in proportion to its own prior level. In our toy system, the cell’s digital promoter state, *P*_i_, is either fully “off” when levels of *S*_*i*_ exceed a threshold, *θ* (identical for all cells and arbitrarily specified to be generally reachable), or otherwise fully “on”. Last, by replacing the gene’s promoter with a light-regulated one that receives virtual promoter “off” and “on” states as inhibiting (red light) or activating (green light) stimuli, respectively, the digital side is interfaced back to the biological side of the system. We tested our hybrid circuits in single cells, and observed oscillating CFP expression over 40 h (Fig. 5c left panel; Supplementary Figs. 5 and 6; Supplementary Movie 5). While the circuits shared similar frequency spectra (Fig. 5c, right panel), variations in individual oscillation lengths shift phases within and between cells. Deterministic behavior of the digital component and low measurement noise suggest that this variability stems largely from the biological side of the circuit.

We asked whether re-specifying the digital component could influence the observed variability. Biological oscillators can synchronize by coupling to extracellular fields, which can either be externally imposed or be a product of the local community^42^. For instance, populations of synthetic bacterial oscillators can synchronize through molecular signals that diffuse between cells, forming weakly-coupled transcriptional networks of oscillators (Fig. 5d, top)^40^. With this biological architecture in mind, we updated our digital component to define a network of connections between the individual bacteria through which the virtualized signal is redistributed (Fig. 5d, bottom). We repeated the experiment while enforcing communication within cyclically connected groups of cells by transferring 20% of each cells’ virtual signal to its nearest neighbors. Improved oscillation peak coincidence and nearly zero period-normalized cross-correlation lags indicate that oscillating cells synchronize (Fig. 5e; Supplementary Fig. 5; Supplementary Movie 6). Given the success of this approach, we explored the behavior of our cells subject to other changes in communication. In our toy system, a freely specifiable transfer matrix *T* (Fig. 5d) defines the connection map, and the strengths and signs of interaction between individual cells.

By changing the signs of off-diagonal transfer matrix values, we generated groups of oscillating cells that either inhibit both neighbors’ accumulation of virtual signal, or stimulate accumulation in one neighbor while inhibiting it in the other. While in our setup this change amounts to a minor change in control software, biological or mechanical implementation of similar operations on bacterial oscillators would be considerably more involved. We tested the new circuits in cyclic, four cell groups. Although we expect the group behaviors to also depend on size and connectivity, patterns in oscillation phases emerge again, but this time with nearest neighbors either a half period, or approximately a quarter period out of phase (Fig. 5f, g; Supplementary Fig. 5; Supplementary Movies 7 and 8).

These simple examples highlight our platform’s potential for straightforward access to diverse, otherwise challenging assays of transcriptional circuits and communication in individual bacteria and populations.

## Discussion

We presented a versatile experimental platform that simultaneously interfaces many individual bacteria with software-defined models in a controlled environment. Very generally, this device represents a step forward in experimental systems built around real-time integration of measurement and control of bacterial cell biology with theoretical models^23,24,26–30,33,43,44^. Directly interweaving “wet” and “dry” components in experiments provides a strong impetus and a ‘test and measurement’ environment for probing predictiveness. By actively testing and adjusting models during experiments, the system could assist in rapid model optimization and facilitate online model inference for single cells^25,45^.

The platform enables quantitative explorations of individual-based traits of bacterial populations through feedback control or digitally specified constraints on gene expression in single cells. The demonstrations above illustrate several directions which can be extended to diverse applications. For instance, distributed behaviors can prepare isogenic populations with incomplete sensory information for stochastic environmental variation^35^. Our device enables exploration of this phenomenon by specifying shapes of and dynamics within expression distributions for populations in specified environments. In such a scenario, cells can even be provided with abilities to artificially “sense” the environment via input from the cells’ software controllers.

In addition, the paradigm of hybrid genetic circuits formed by interfacing biological and digitally-modeled parts allows biological systems to be characterized under constraint by virtual circuit parts. Analogous to unit testing in software development, this capacity could ease challenges in piecewise debugging of genetic circuits, and in situ development and optimization of synthetic circuits within cell factories^20,22^. In the latter, in contrast to entirely wet or entirely theoretical approaches, real impacts of virtual components such as sensors could be approximated even before biological versions are engineered, thus speeding up development.

Finally, by specifying hybrid circuits that allow information to flow between individual cells, the system enables new kinds of experiments on how collective behaviors of small groups of bacteria respond to community and environment-dependent changes in cell interactions^1,9,37^. Assays of behaviors that respond to digitally defined cell interactions and perturbations (e.g., virtual diffusion or cell arrangement) could assist in exploring interaction-targeted programs for stabilizing, disrupting, or otherwise controlling natural and artificial bacterial communities^11,13,17,18,46^.

Individual and group behaviors of microbes are hard to approach fully through simulation alone or simply through biological assay alone. By virtualizing parts of biological systems, our platform achieves benefits of both methods by allowing straightforward specifiability of a virtualized part within its full biological context. Importantly, transplanting digitally specified components into biological systems can extend the explorable space of circuits and behaviors to and even beyond what is biologically possible^47^. On the one hand, for characterizing actual genetic circuits within and between cells, the platform can guide (although not replace) traditional fully biological analyses. On the other hand, its reduced constraints allow us to ask “What if?” questions and to create, perturb, and study approximations to otherwise inaccessible biological systems. Following on the proofs of concept above, our platform can thus facilitate new modes of exploring behaviors of populations and other complex bacterial systems.

## Methods

### Bacterial strains and plasmids

See Table 2.

**Table 2:** Bacterial strains and plasmids

Name	Genotype	Source
pSR43.6	p15A ori, spR, ccaS, ho1-pcyA	Ref. ^31^
pSR58.6	colE1 ori, cmR,ccaR, P_cpcG2-172_sfGFP	Ref. ^31^
pSR58.6_cerulean	pSR58.6 ΔsfGFP::ceruleanCFP	This work
TB201	MG1655 Pr_venusYFP	This work
CR138	TB201 /pSR43.6, pSR58.6_cerulean	This work
JW1908	BW25113 ΔfliC769::kan	KEIO collection^56^
CR141	TB201 Δ769fliC::frt	This work
CR145	CR141 /pSR43.6, pSR58.6_cerulean	This work

### Imaging and projection system

Our system integrates image data acquisition and processing, cell stimulation, and environmental regulation. All physical device control and data processing is executed through custom software in MATLAB (version 2014b, with statistics, image processing, distributed computing toolboxes) using MicroManager^48^, SUNDIALS^49^, mexOpenCV^50^, and java packages (Supplementary Software).

Cell growth and expression data are derived from images collected with a motorized inverted microscope (Body: Olympus IX83, Stage:Märzhäuser, Objective:Olympus UPLSAPO100XOPH, Camera: Hamamatsu Orca Flash4.0v2, LED-based fluorescence illuminator: Lumencor SpectraX) in the CFP (x438/29,m483/22) and YFP (x513/22,m543/22) fluorescence channels.

Light stimuli are simultaneously delivered to cells in a field of view using a variant of a custom modified LCD projector (Supplementary Fig. 1)^51^. The projector (Panasonic PT-AE6000E) iris is disabled and lamp replaced by 530 and 660 nm LED sources (Thorlabs, M530L3, M660L3, LEDD1B). The outer, projection, lens is removed and the image projected through the zoom lens is coupled via a field lens (Thorlabs AC508-100-A-ML, *f* = 100 mm) into the rear port of the microscope, through a tube lens (Olympus U-TLU), and via a 50/50 beamsplitter (Thorlabs BSW10R, TOFRA filter cube slider) through the objective and onto the field of view (Supplementary Figs. 7 and 8). Projector position is adjusted to bring the camera and projector focal planes into alignment, and sub-micron corrections between the focal planes to be used during the experiment are determined, per channel, at its outset.

Experimental temperature is regulated within a custom-built opaque, temperature-controlled microscope enclosure via recirculating air heater (controller: Cal controls CAL3200). Media delivery is regulated by a pair of syringe pumps (WPI, Alladin-1000).

### Imaging, image processing, and cell stimulation loop

During operation, software-based focus (modified micro-manager oughtafocus function) is determined at each location/time-point using reflective imaging (475/34 nm) of PDMS-glass interfaces. A focused reflection image is used for a phase correlation-based estimate of *x* axis and *y* axis corrections to stage jitter. Fluorescent images are acquired, corrected for a small, slowly varying additive offset, and divided by previously-obtained, normalized calibration images of a uniform fluorescent field^52^ (10% Fluorescein 0.1%NaHCO_3_) to correct for fluorescence shading variation. The images are then spatially registered and fluorescence-based expression estimates (constitutive, controlled channels) are extracted for the individual mother cells under control as the 97th percentile pixel intensity within a pre-specified bounding box at each cell’s image location (Fig. 1, dashed box). In the present experiments, this detection region is fixed at 40 × 80 pixels (~13.5 μm^2^), of which the 3% above the extracted value (96 pixels) sums to ~0.4 μm^2^. To avoid injecting large errors into closed loops, this method was selected for its lack of cell segmentation errors and robustness to large variations in cell size, position and fluorescence. The constitutive fluorescence images are separately segmented to derive sizes of mother cells, and growth rates are estimated via a moving average of differences in log_2_(cell length), excluding extreme outliers resulting from cell division events and transient segmentation errors. This per-cell data are passed to experiment-dependent software controllers that update cell state estimates and determine the subsequent activation (~535 nm) or deactivation (~670 nm) light stimuli to be delivered to each cell (Supplementary Methods).

The list of per-cell stimuli is converted to red and green boxes in an RGB image, overlying the positions of their corresponding cells (typically covering the terminal ~11 μm of channel, e.g., position of dashed yellow box in Supplementary Fig. 2). The image is then spatially transformed to register projector to camera image planes (openCV function cv.warpPerspective, using homographies determined at the experiment outset with projected chessboards and the functions cv.findChessboardCorners and cv.findHomography). Shading corrections (low-pass frequency filtered images of reflected flat field projections, generated at experiment setup) are applied to each color channel. The image is then projected onto the field of view and cells illuminated for 10 s with ~10.5 red or 7.6 mW cm^−2^ green light (with contrast ratios relative to dark LCD panels, of 252 and 361, respectively; crosstalk between channels is <1%).

Although our setup also permits control via mixed red–green and intermediate level light dosing of the CcaSR light system (as employed by Olson et al.^27^), we opted for a pulse-width modulation (PWM) type scheme^53^ and brief monochromatic stimuli to facilitate rapid cycling through multiple fields of view. Six-minute intervals were chosen to maximize cell numbers per experiment and computational time per control loop while maintaining sufficiently fast control dynamics relative to expression and dilution timescales (Supplementary Methods).

To reduce crosstalk with the optogenetic systems, cell exposures to imaging wavelengths are minimized and kept invariant across all experiments. In addition, the interval between exposing cells to imaging wavelengths and setting their new optogenetic system state is kept to a minimum by immediately following data acquisition with application of new stimuli. When using controllers that require significant computational time to generate new light sequences, those stimuli calculated during the previous loop are applied before computing those to apply in the next.

### Microfluidic devices

Microfluidic mother machines allow straightforward, stable culture and observation of individual cells for hundreds of generations, in contrast to microscope culture techniques which do not actively remove progeny during growth. Our devices (23 µm × 1.3 µm × 1.3 µm (*l*, *w*, *h*) growth channels with 5 µm spacing along a split media trench, Supplementary Fig. 2) are fabricated by curing degassed polydimethylsiloxane (Sylgard 184, 1:10 catalyst:resin) against epoxy replicate master molds produced from primary wafer-molded devices^54,55^. Cured PDMS bulk is peeled from the molds and trimmed as appropriate, input and output ports are punched with electropolished 18ga luer stubs. The PDMS bulk and a clean cover slip are rinsed with 100% isopropanol, blown dry, baked on a hotplate at ~125 °C for 15 min. They are then cooled, and the surfaces to be bonded exposed to air plasma (Harrick PDC-002 plasma cleaner, medium power) for 1 min, and then brought gently into contact. The bonded devices are left at room temperature for 15 min, post baked for 1–2 h at 80 °C, and then stored until use. Polyethylene tubing (Instech, BTPE-50) is press-fitted onto 22ga luer stubs and cannulae (Instech) on opposite ends for coupling to media supplies and waste, and to the devices, respectively.

### Experiment setup and conditions

A frozen glycerol cell stock is thawed from −80 °C, diluted 1:100 into 5 ml fresh LB containing 0.01% Tween20, with 20 µg ml^−1^ Chloramphenicol and 100 µg ml^−1^ Spectinomycin to maintain plasmids, and incubated for 6–7 h at 37 °C. The experimental apparatus is initialized, prewarmed and equilibrated, and the microfluidic device flushed for 1 min with 0.01% Tween20 followed by air. The device is mounted to the microscope stage to warm and verify integrity. The grown cell culture is centrifuged at 4000×*g* for 4 min, and the pellet resuspended in a few µl supernatant and injected into the device by pipette. Once filling of the growth channels with cells is confirmed under the microscope, media supply and waste tubes are fitted to the device and running media (LB, 0.4% glucose, 0.01% Tween20) is flowed through the device at 4 ml h^−1^ for 1 h, and 1.5−2.0 ml h^−1^ thereafter. The experiment control software is engaged (Supplementary Fig. 9). Experiment calibration, providing per-channel camera and projector offsets from the PDMS-glass interface focal plane, projector-camera image transforms, and projector shading correction are performed. For each control location on the chip, measurement areas for individual cells are specified (typically, by a 2.6 × 5.2 μm box at the end of a growth channel), and a software controller/target program is associated with each. Once all control locations have been populated and the system begins to acquire data and stimulate the cells, it runs automatically until the conclusion of the experiment. For experiments involving media switching, a stopcock and syringe pump flows are adjusted at the appropriate time (0.3 ml media plug between the stopcock and cells is replaced after approximately two 6-min cycles).

### Media

LB containing 0.01% Tween20, 100 µg ml^−1^ Spectinomycin, and 20 µg ml^−1^ Chloramphenicol is used for strain preculture and plasmid maintenance preceding insertion of cells into the device. Running media (LB, 0.01% Tween20, 0.4% Glucose) is used thereafter. For doxycycline perturbations, a 1 µg ml^−1^ stock solution of doxycycline is diluted in running media to a final experimental concentration, and maintained at 23 °C in the dark for the duration of the experiment.

### Cell classification and invalidation

Over time, bacterial cells in mother machine devices may filament and/or halt growth^14^. Optogenetic systems are also subject to mutational dysfunction and plasmid loss from cells. Finally, cells can temporarily shift from focal locations or escape from channels entirely. In contrast to experimental setups that sample new cells from batch culture at each time point, such pathological cells and locations persist indefinitely in microfluidic mother machines without being outgrown or replaced. They must therefore be algorithmically classified and invalidated to avoid corrupting data of otherwise normal populations or from injecting spurious signals into closed loops.

To remove such pathological cells and locations from our experiments, we run a classification algorithm in which the mother cells in our device are automatically evaluated for continuous presence, growth, and maintenance of the optogenetic system. We use our cells’ constitutively expressed YFP to verify cell presence at control locations and fluorescence measurement quality (YFP signal loss or excessive noisiness invalidates cells/locations). In addition, YFP images are segmented to extract cell shapes and invalidate non-growing cells whose smoothed elongation rate has fallen below a minimum threshold. Besides complete growth arrest, loss of plasmids from our system can occur, resulting in either: (i) a reduced YFP level due to a substantially increased growth and dilution rate (pSR43.6) or (ii) complete, extended loss of responsiveness and CFP signal (pSR58.6_cerulean). These are used to automatically classify cells as invalid when locally time-averaged YFP level or controller-estimated responsiveness fall below set thresholds. In general, cells that are ruled invalid in one test are eliminated for the remainder of the experiment.

If cell classification is used for post-experiment analysis, the data of invalidated cells is truncated to 150 min before the threshold violation to reduce inclusion of post-failure, pre-invalidation data. However, where cells are directly coupled through a controller, as in our pCL control experiments, then cell classification and invalidation is implemented and cells are removed in real time from the population contributing to control (although they can continue to receive light stimuli). In such cases, infrequently appearing cells that increase to extremely high levels of CFP fluorescence before being invalidated remain an impediment to good control. Therefore, in these cases, a second algorithm is used to rapidly identify such cells and block their contribution to the population until they are invalidated (Supplementary Methods, section 2.4).

### Data availability

Strains and data are available from the authors upon request. Custom scripts for the described setup are available as Supplementary Software.

## Electronic supplementary material

Supplementary Information

Description of Additional Supplementary Files

Supplementary Movie 1

Supplementary Movie 2

Supplementary Movie 3

Supplementary Movie 4

Supplementary Movie 5

Supplementary Movie 6

Supplementary Movie 7

Supplementary Movie 8

Supplementary Software

## References

[1] Widder S (2016). Challenges in microbial ecology: building predictive understanding of community function and dynamics. ISME J..

[2] Hughes DT, Sperandio V (2008). Inter-kingdom signalling: communication between bacteria and their hosts. Nat. Rev. Microbiol..

[3] Vetsigian K, Jajoo R, Kishony R (2011). Structure and evolution of streptomyces interaction networks in soil and in silico. PLoS Biol..

[4] Wintermute EH, Silver PA (2010). Dynamics in the mixed microbial concourse. Genes Dev..

[5] Shank EA, Kolter R (2011). Extracellular signaling and multicellularity in *Bacillus subtilis*. Curr. Opin. Microbiol..

[6] Ackermann M (2015). A functional perspective on phenotypic heterogeneity in microorganisms. Nat. Rev. Microbiol..

[7] Claessen D, Rozen DE, Kuipers OP, Søgaard-Andersen L, van Wezel GP (2014). Bacterial solutions to multicellularity: a tale of biofilms, filaments and fruiting bodies. Nat. Rev. Microbiol..

[8] Vlamakis H, Aguilar C, Losick R, Kolter R (2008). Control of cell fate by the formation of an architecturally complex bacterial community. Genes Dev..

[9] Straight PD, Kolter R (2009). Interspecies chemical communication in bacterial development. Annu. Rev. Microbiol..

[10] Fauvart M, De Groote VN, Michiels J (2011). Role of persister cells in chronic infections: clinical relevance and perspectives on anti-persister therapies. J. Med. Microbiol..

[11] Rutherford ST, Bassler BL (2012). Bacterial quorum sensing: its role in virulence and possibilities for itscontrol. Cold Spring Harb. Perspect. Med..

[12] Ackermann M (2008). Self-destructive cooperation mediated by phenotypic noise. Nature.

[13] Grandclément C (2016). Quorum quenching: role in nature and applied developments. FEMS Microbiol. Rev..

[14] Wang P (2010). Robust growth of *Escherichia coli*. Curr. Biol..

[15] Gefen O, Balaban NQ (2009). The importance of being persistent: heterogeneity of bacterial populations under antibiotic stress. FEMS Microbiol. Rev..

[16] Vega NM, Allison KR, Khalil AS, Collins JJ (2012). Signaling-mediated bacterial persister formation. Nat. Chem. Biol..

[17] Chuang JS (2012). Engineering multicellular traits in synthetic microbial populations. Curr. Opin. Chem. Biol..

[18] Kong W, Celik V, Liao C, Hua Q, Lu T (2014). Programming the group behaviors of bacterial communities with synthetic cellular communication. Bioresour. Bioprocess..

[19] Guet CC, Elowitz MB, Hsing W, Leibler S (2002). Combinatorial synthesis of genetic networks. Science.

[20] Kwok R (2010). Five hard truths for synthetic biology: can engineering approaches tame the complexity of living systems? Roberta Kwok explores five challenges for the field and how they might be resolved. Nature.

[21] Potvin-Trottier L, Lord ND, Vinnicombe G, Paulsson J (2016). Synchronous long-term oscillations in a synthetic gene circuit. Nature.

[22] Holtz WJ, Keasling JD (2010). Engineering static and dynamic control of synthetic pathways. Cell.

[23] Milias-Argeitis A (2011). In silico feedback for in vivo regulation of a gene expression circuit. Nat. Biotechnol..

[24] Milias-Argeitis A, Rullan M, Aoki SK, Buchmann P, Khammash M (2016). Automated optogenetic feedback control for precise and robust regulation of gene expression and cell growth. Nat. Commun..

[25] Ruess J, Parise F, Milias-Argeitis A, Khammash M, Lygeros J (2015). Iterative experiment design guides the characterization of a light-inducible gene expression circuit. Proc. Natl Acad. Sci. USA.

[26] Uhlendorf J (2012). Long-term model predictive control of gene expression at the population and single-cell levels. Proc. Natl Acad. Sci. USA.

[27] Olson EJ, Hartsough LA, Landry BP, Shroff R, Tabor JJ (2014). Characterizing bacterial gene circuit dynamics with optically programmed gene expression signals. Nat. Methods.

[28] Menolascina F (2014). In-vivo real-time control of protein expression from endogenous and synthetic gene networks. PLoS Comput. Biol..

[29] Melendez J (2014). Real-time optogenetic control of intracellular protein concentration in microbial cell cultures. Integr. Biol..

[30] Toettcher JE, Gong D, Lim WA, Weiner OD (2011). Light-based feedback for controlling intracellular signaling dynamics. Nat. Methods.

[31] Schmidl SR, Sheth RU, Wu A, Tabor JJ (2014). Refactoring and optimization of light-switchable *Escherichia coli* two-component systems. ACS Synth. Biol..

[32] Hirose Y, Shimada T, Narikawa R, Katayama M, Ikeuchi M (2008). Cyanobacteriochrome CcaS is the green light receptor that induces the expression of phycobilisome linker protein. Proc. Natl Acad. Sci. USA.

[33] Fiore G, Perrino G, di Bernardo M, di Bernardo D (2016). In vivo real-time control of gene expression: a comparative analysis of feedback control strategies in yeast. ACS Synth. Biol..

[34] Davidson CJ, Surette MG (2008). Individuality in bacteria. Annu. Rev. Genet..

[35] Kussell E (2005). Bacterial persistence: a model of survival in changing environments. Genetics.

[36] Walsh, C. *Antibiotics: Actions, Origins, Resistance* (ASM Press, Washington, D.C., 2003).

[37] Aminov RI (2009). The role of antibiotics and antibiotic resistance in nature. Environ. Microbiol..

[38] Deris JB (2013). The innate growth bistability and fitness landscapes of antibiotic-resistant bacteria. Science.

[39] Novák B, Tyson JJ (2008). Design principles of biochemical oscillators. Nat. Rev. Mol. Cell Biol..

[40] Danino T, Mondragón-Palomino O, Tsimring L, Hasty J (2010). A synchronized quorum of genetic clocks. Nature.

[41] Stricker J (2008). A fast, robust and tunable synthetic gene oscillator. Nature.

[42] Strogatz SH, Stewart I (1993). Coupled oscillators and biological synchronization. Sci. Am..

[43] Tschirhart T (2017). Electronic control of gene expression and cell behaviour in *Escherichia coli* through redox signalling. Nat. Commun..

[44] Rullan, M. et al. Optogenetic single-cell control of transcription achieves Mrna tunability and reduced variability. Preprint at 10.1101/142893 (2017).

[45] Zechner C, Unger M, Pelet S, Peter M, Koeppl H (2014). Scalable inference of heterogeneous reaction kinetics from pooled single-cell recordings. Nat. Methods.

[46] Keller L, Surette MG (2006). Communication in bacteria: an ecological and evolutionary perspective. Nat. Rev. Microbiol..

[47] Jacob, F. *The Possible and the Actual* (Pantheon Books, New York, 1982).

[48] Edelstein AD (2014). Advanced methods of microscope control using μManager software. J. Biol. Methods.

[49] Hindmarsh AC (2005). SUNDIALS: suite of nonlinear and differential/algebraic equation solvers. ACM Trans. Math. Softw. TOMS.

[50] Yamaguchi, K. mexOpenCV. https://github.com/kyamagu/mexopencv (2014).

[51] Stirman JN, Crane MM, Husson SJ, Gottschalk A, Lu H (2012). A multispectral optical illumination system with precise spatiotemporal control for the manipulation of optogenetic reagents. Nat. Protoc..

[52] Model MA, Burkhardt JK (2001). A standard for calibration and shading correction of a fluorescence microscope. Cytometry.

[53] Davidson EA, Basu AS, Bayer TS (2013). Programming microbes using pulse width modulation of optical signals. J. Mol. Biol..

[54] Estévez-Torres, A., Yamada, A. & Wang, L. An inexpensive and durable epoxy mould for PDMS. Chips and Tips (Lab on a Chip) http://blogs.rsc.org/chipsandtips/2009/04/22/an-inexpensive-and-durable-epoxy-mould-for-pdms/ (2009).

[55] Bergmiller T (2017). Biased partitioning of the multidrug efflux pump AcrAB-TolC underlies long-lived phenotypic heterogeneity. Science.

[56] Baba T (2006). Construction of *Escherichia coli* K‐12 in‐frame, single‐gene knockout mutants: the Keio collection. Mol. Syst. Biol..

