# Supplementary material for: Shaping bacterial population behavior through computer-interfaced control of individual cells
Source: Nat Commun. 2017 Nov 16;8:1535. doi: 10.1038/s41467-017-01683-1 (PMC5688142; doi:10.1038/s41467-017-01683-1)
Supplement: Supplementary file 1 — Supplementary Information [file 41467_2017_1683_MOESM1_ESM.pdf]

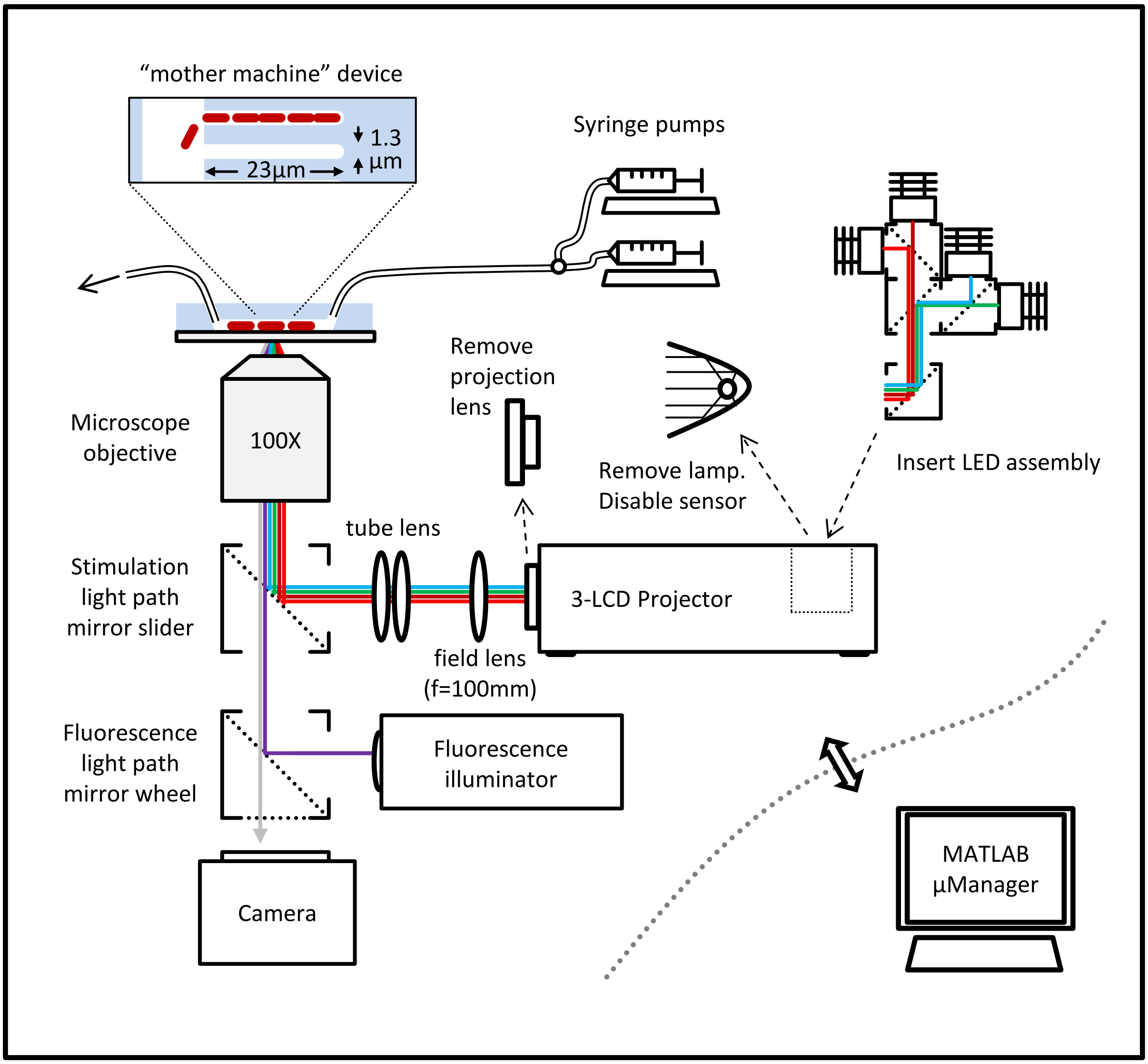

Supplementary Figure 1 | Single cell measurement and control platform

Cells are grown within a microfluidic "mother machine" device (Supplementary Fig. 2), fed by media from two syringe pumps (WPI, Alladin 1000). The device is on an incubated (custom incubator not shown) automated microscope (Olympus, IX83), modified with a mirror slider (TOFRA) and tube lens to provide an additional light path for stimulation images. A 3-LCD overhead projector (Panasonic PT-AT6000E) is modified by removing the projection lens (leaving the zoom lens in place), and replacing the projector lamp with a custom-built LED illuminator with four independent channels. The projector lamp safety circuit is disabled by connecting its optocoupler leads. Images focused a few cm in front of the modified projector are relayed through a field lens and into the stimulation light path of the microscope. All automated equipment is directly controlled by MATLAB and the micro-manager api.

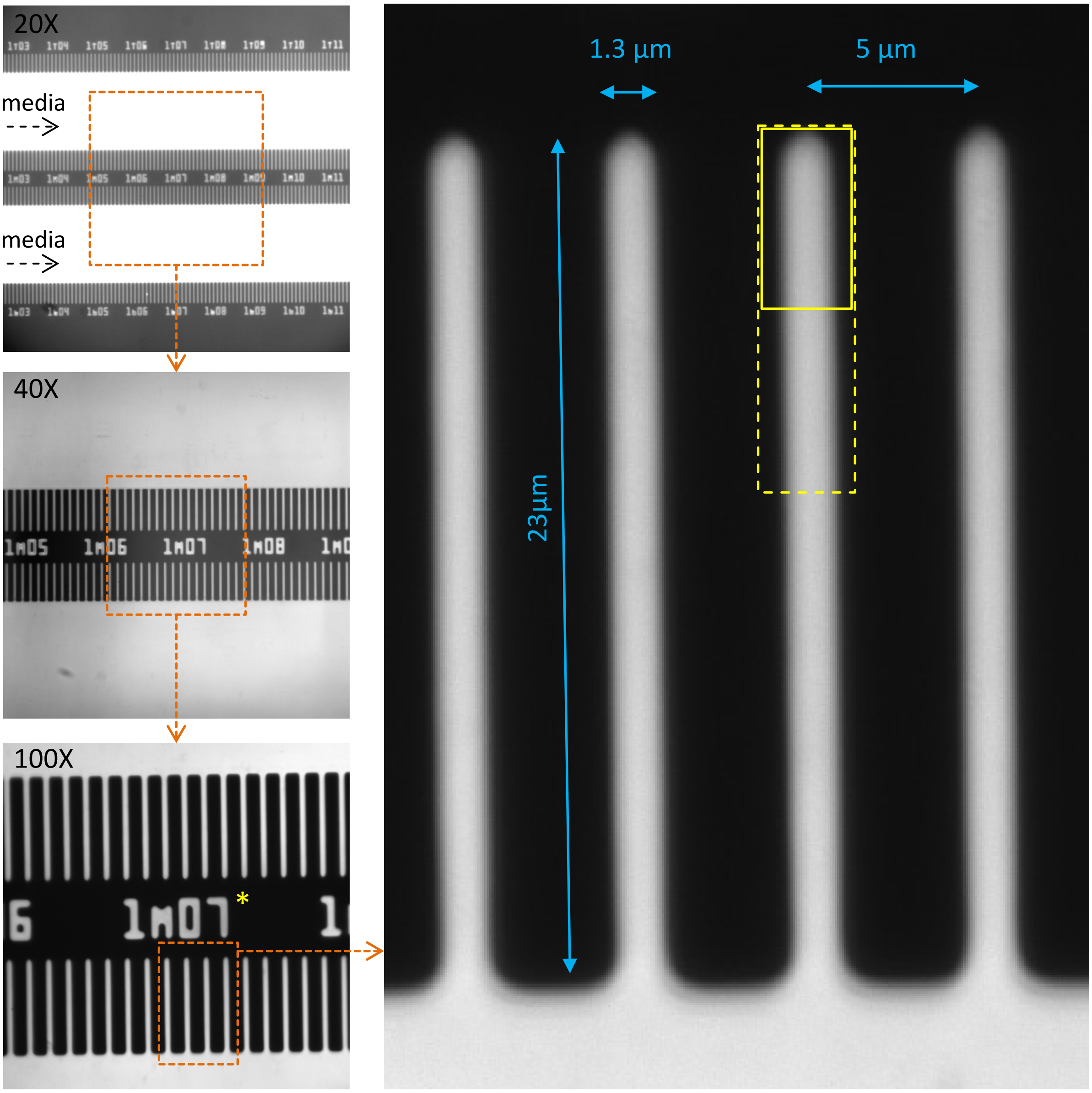

Supplementary Figure 2 | Microfluidic mother machine, imaged at glass interface
Lighter and darker regions are channels and PDMS bulk, respectively. Media flows left to right through the upper and lower trenches (top left image). Cells grow constrained within fine, dead end channels (approximately 1.3µm X 23µm) intersecting the main trench every 5µm. Typical detection (yellow, solid) and stimulation (yellow, dashed) regions for a mother cell are illustrated. Device location numbers (yellow asterisk) provide useful features for calculating fine registrations of data and projection images with cell locations.

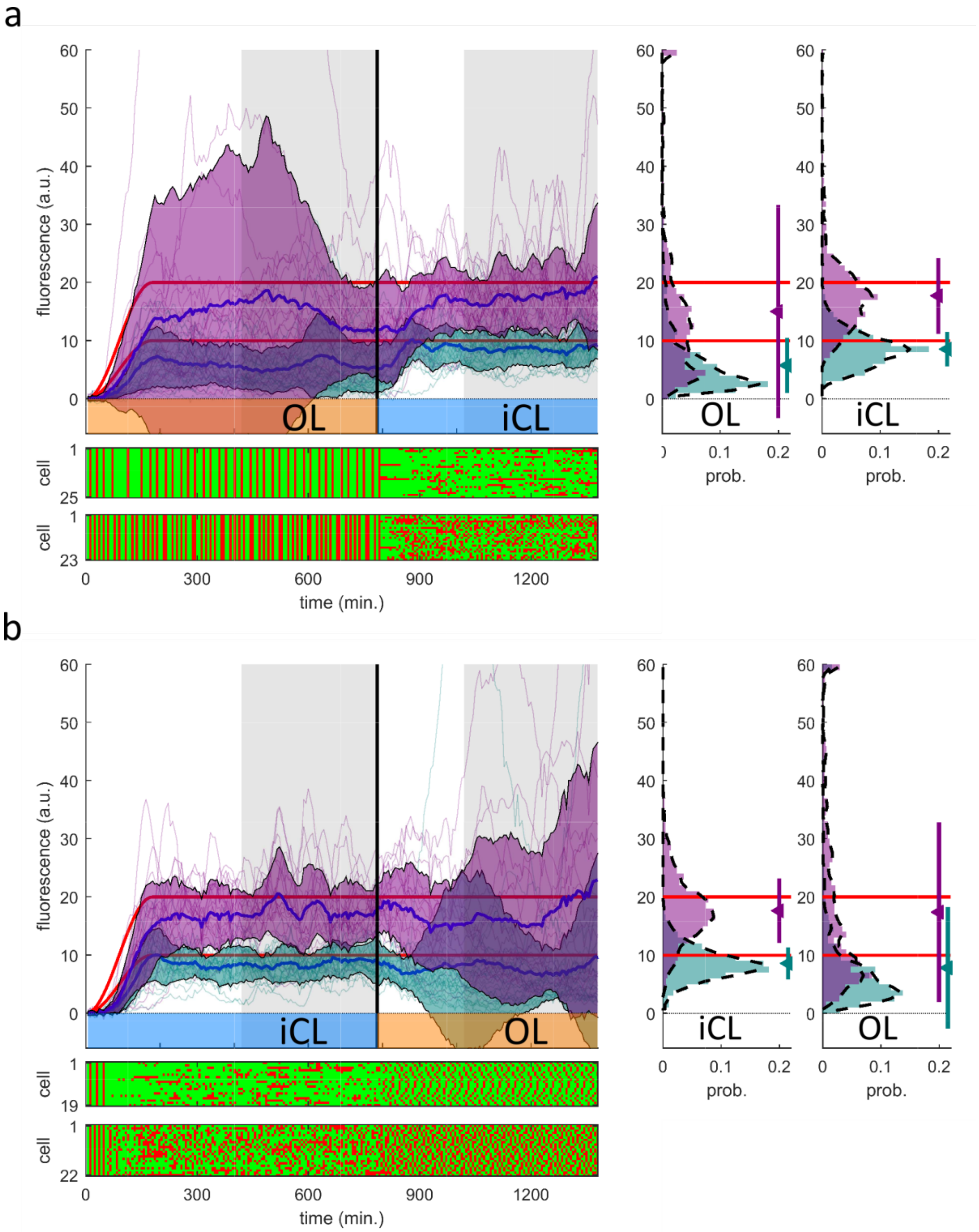

Supplementary Figure 3 | Improved resolution of subpopulations with multiple expression targets by individual closed loop (iCL) control either when following or preceding open loop (OL) control
Mean CFP trajectories +/- one standard deviation (blue lines and purple, teal-shaded regions, mean trajectories smoothed with 1 hour moving average) for cells controlled towards fluorescence targets (red lines) of either 10 a.u. or 20 a.u. Within the same experiment, cell control modes are switched from either: **(a)** open loop (orange labelled time interval) to individual closed loop (blue interval) mode, or **(b)** individual closed loop to open loop, at 780 min (black vertical line). Individual light stimulation sequences (green: activation, red: repression) for the valid cells (Methods) at experiment end targeted to 20 a.u. (upper sequences) and 10 a.u. (lower sequences) are displayed below the trajectory plots. To the right, probability distributions (purple, teal shaded with dashed smoothed envelopes), means (triangles), and variation (error bars +/- 1 s.d.) of CFP levels in the differently targeted groups during open and individual closed loop control regimes (OL, iCL grey shaded time intervals, respectively) are shown. Distribution overlaps are reduced under iCL control. All data are derived from the same experiment using equally-sized subsets of initialized cells per field of view.

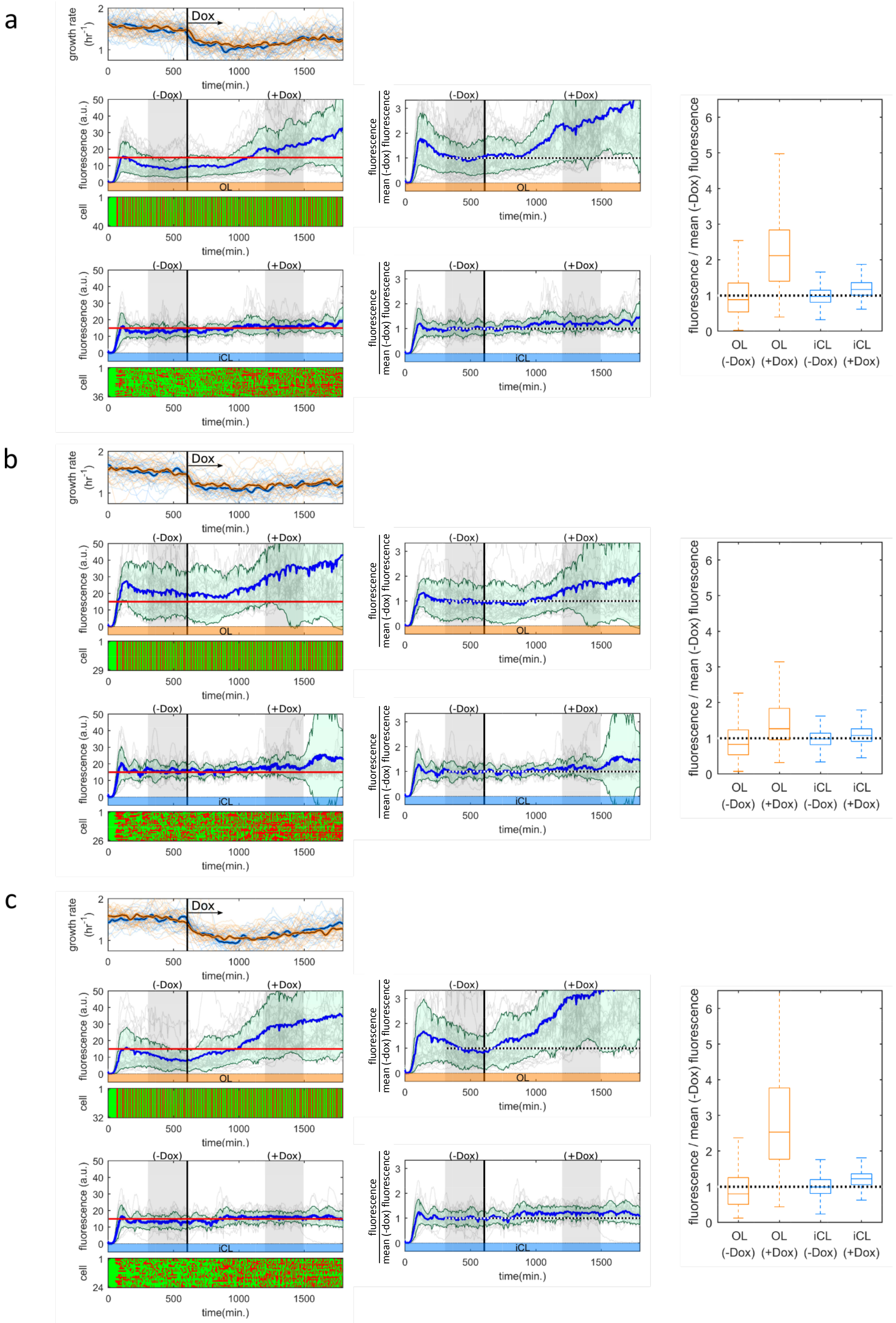

a
b
c
growth rate (hr-1)
Dox
time(min.)
(-Dox)
(+Dox)
fluorescence (a.u.)
OL
iCL
cell
fluorescence
mean (-dox) fluorescence
fluorescence / mean (-Dox) fluorescence
OL (-Dox)
OL (+Dox)
iCL (-Dox)
iCL (+Dox)

Supplementary Figure 4 | iCL controlled CFP expression is less impacted than OL controlled expression by exposure to growth inhibiting levels of doxycycline antibiotic

a-c. Three replicate experiments in which bacteria under open loop (OL) and individual closed loop (iCL) control to a constant target are exposed to 0.6 ug/ml doxycycline added to the culture media at 600 min (vertical black line). Total numbers of growing cells with functioning optogenetic systems at experiment completion = 76,65,56. For each experiment, OL and iCL controlled cells are exposed to maximally similar environments by assigning the controllers to equal subsets of cells per field of view. The data from Figure 4 is repeated here (**a,** left,right panels) for comparing normalized trajectories. For each experiment, growth rates (Left top panels, individual: light lines; mean: dark lines) of cells under OL control (orange), and iCL control (light blue) decline following addition of 0.6 ug/ml doxycycline. Individual and mean CFP fluorescence trajectories (grey, blue lines respectively; green shaded regions = mean +/- 1 s.d.) are shown for cells tracking a constant target (15 a.u.) under OL control (Left, middle panel) or iCL control (Left, lower panel). Light stimulation sequences are displayed below the fluorescence plots. Pre-antibiotic normalized CFP trajectories (grey:individual, blue:mean, green shaded regions = mean +/- 1 s.d.) of OL controlled (center, upper panel) and iCL controlled (center, lower panel) cells are shown. For each controller, CFP trajectories are divided by their population mean during the (–Dox) shaded interval, 0-5 hours prior to doxycycline perturbation. Box plot distributions (right panel) show relative perturbations of CFP fluorescence in OL- and iCL-controlled populations following doxycycline addition (measured during (+Dox) interval, 10-15 hours after doxycycline is added, relative to mean expression by the same cells during the (-Dox) interval).

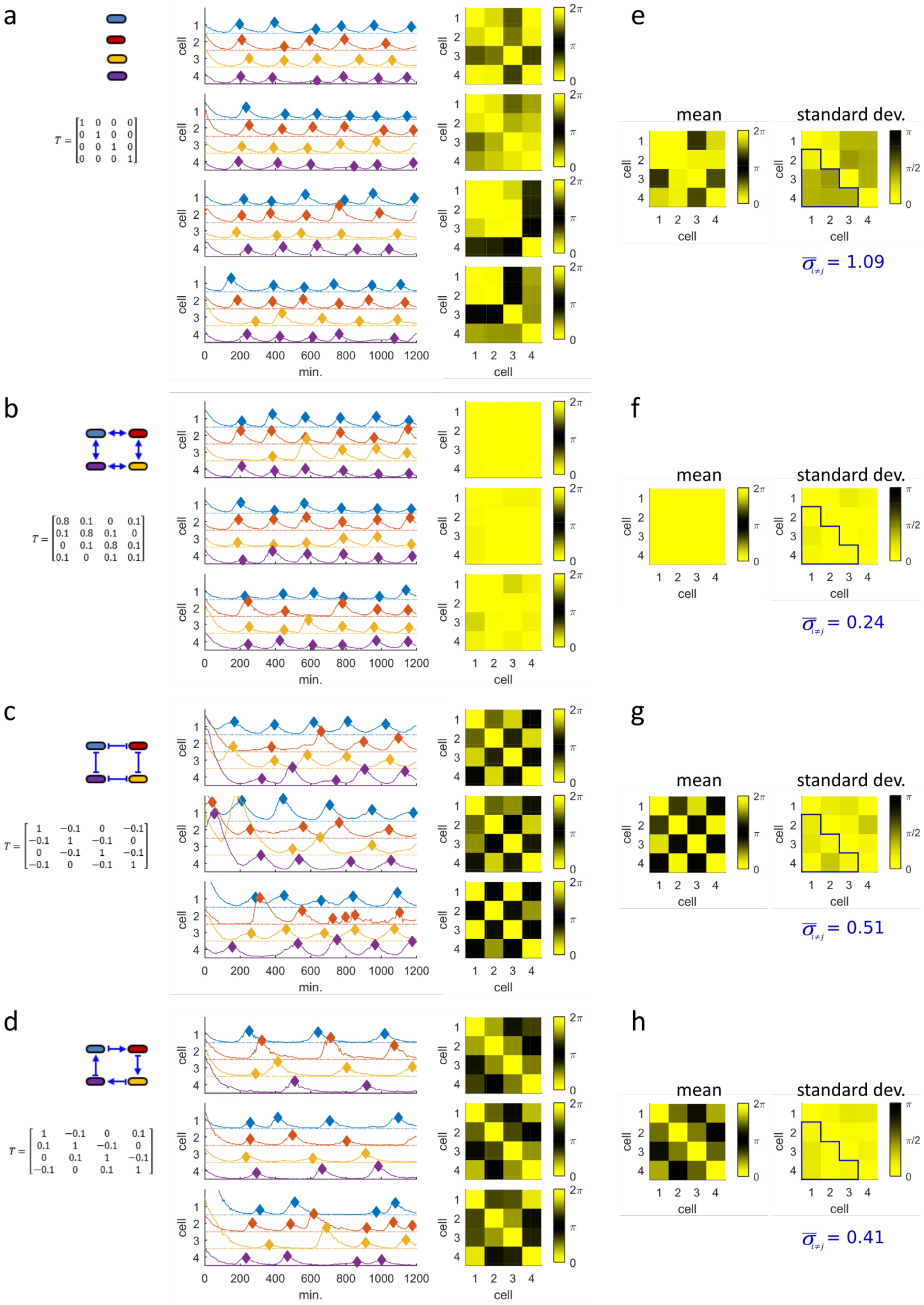

a
b
c
d
e
f
g
h
T = [1 0 0 0; 0 1 0 0; 0 0 1 0; 0 0 0 1]
T = [0.8 0.1 0 0.1; 0.1 0.8 0.1 0; 0 0.1 0.8 0.1; 0.1 0 0.1 0.1]
T = [1 −0.1 0 −0.1; −0.1 1 −0.1 0; 0 −0.1 1 −0.1; −0.1 0 −0.1 1]
T = [1 −0.1 0 0.1; 0.1 1 −0.1 0; 0 0.1 1 −0.1; −0.1 0 0.1 1]
cell
min.
0
200
400
600
800
1000
1200
2π
π
π/2
mean
standard dev.
σ̄i≠j = 1.09
σ̄i≠j = 0.24
σ̄i≠j = 0.51
σ̄i≠j = 0.41

Supplementary Figure 5 | CFP trajectories and phase lags for 4-member interacting networks of hybrid oscillators

a-d. CFP fluorescence trajectories (left) and cross correlation phase lags (right) of four-member sets of single-cell hybrid oscillators over 20 hours (replicates are from the same experiment). A digitally-specified coupling matrix, T, links the cells into networks with (**a**) no interactions ($r = 3, d = 0.2, \theta = 60$), (**b**) positive interactions ($r = 3, d = 0.2, \theta = 60$, for every cell, $0.1S$ is transferred to the nearest neighbors, per interval), (**c**) negative interactions ($r = 3, d = 0.2, \theta = 60$, for every cell, $0.1S$ is removed from the nearest neighbors per interval), (**d**) asymmetric interactions ($r = 3, d = 0.16, \theta = 60$, for every cell, $0.1S$ is respectively added and removed from the left- and right-hand nearest neighbors, per interval). Filled diamonds in the leftmost panels denote expression peaks of the trajectories (fit after local smoothing), for comparing oscillation timing between cells. 0-20 hour data from Figure 5 is included in the uppermost plot for each network type. **e-h.** Circular means (left) and standard deviations (right) of phase lags over the replicate four-cell groups shown in **a-d** are displayed along with the average standard deviation over all non-identical cell locations on the networks (blue outline in right panels).

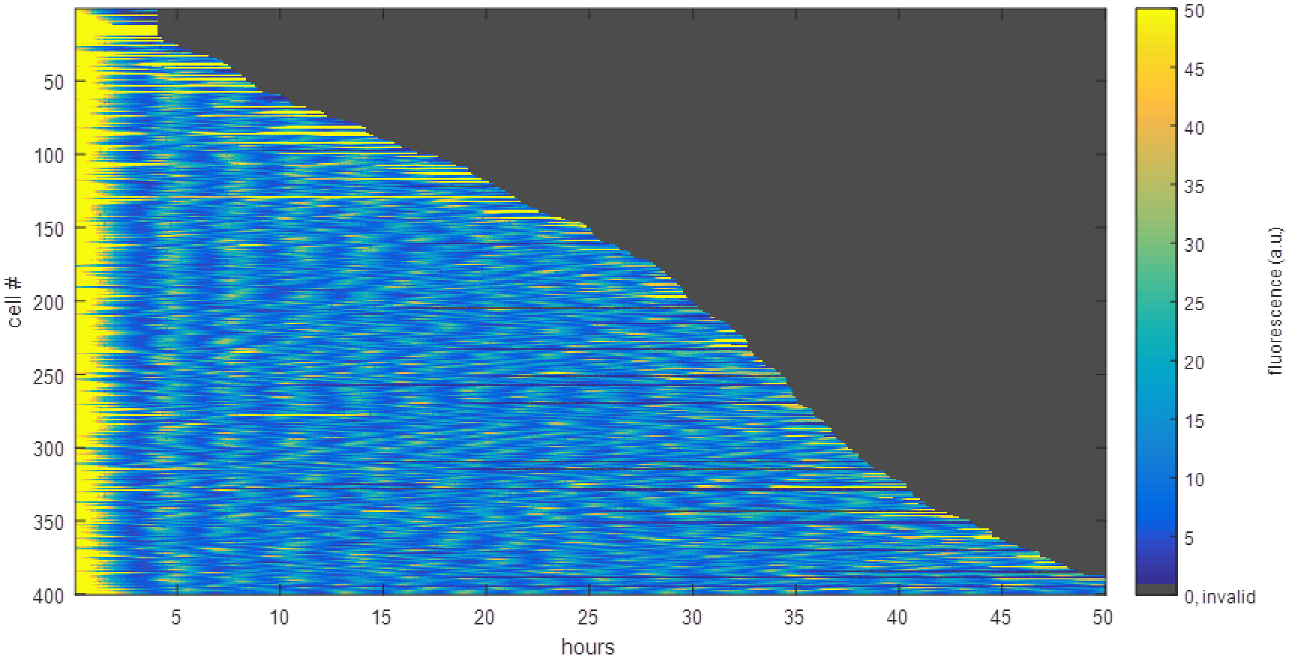

Supplementary Figure 6 | Online invalidation of cells operating as uncoupled hybrid oscillators

CFP fluorescence trajectories of 400 cells, ordered by duration of automatic classification of validity. Individual cells are automatically and permanently eliminated from the valid population during a single, 50 hour experiment when moving averages cross thresholds for high measurement noise, filamentation or very slow growth, low expression of the constitutive reporter, and very low estimated responsiveness. The cells are protected for the first four hours.

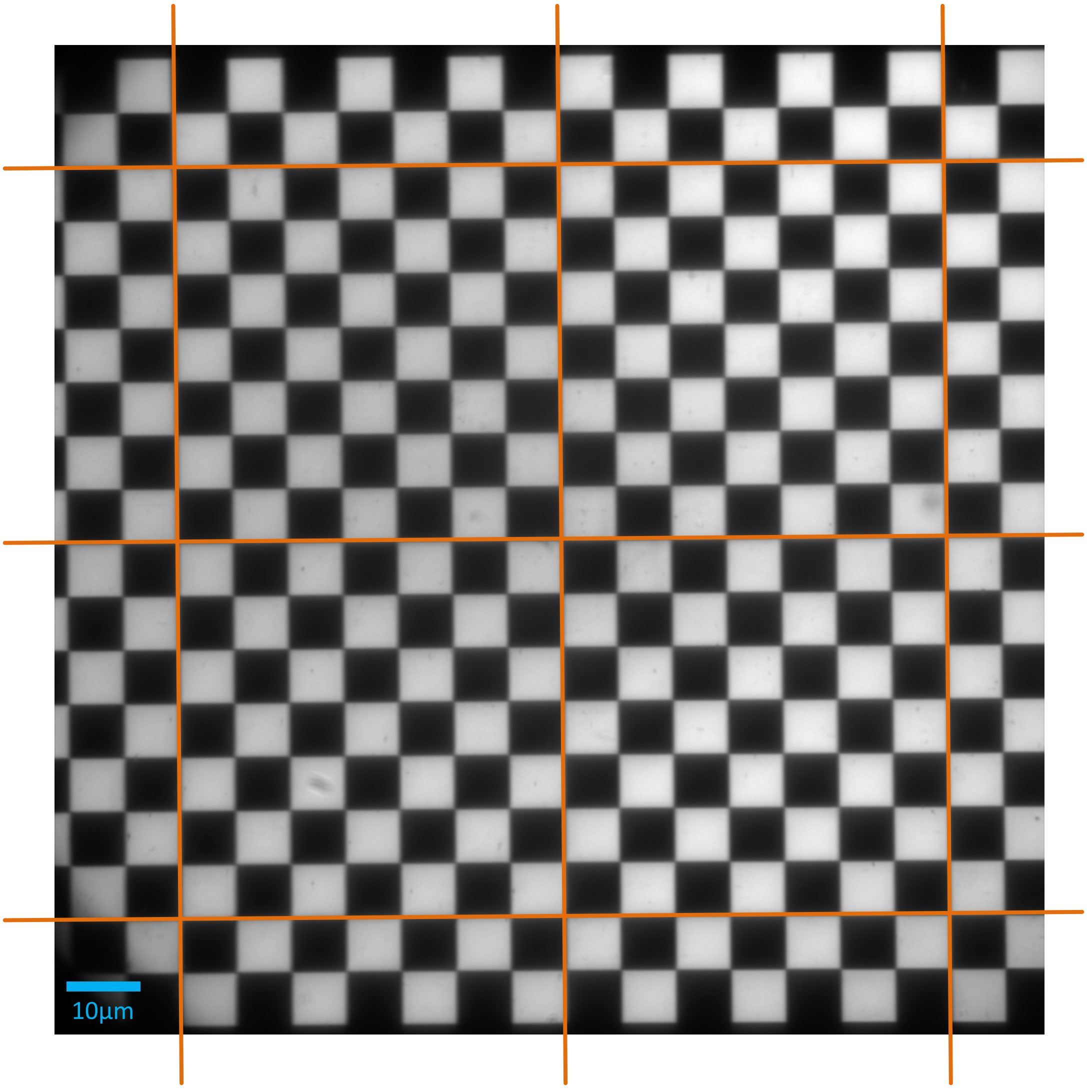

Supplementary Figure 7 | Projected image shows little radial distortion
Full camera chip image (~133µm/side) of reflection of projected checkerboard pattern without warping or shading corrections. Alignment of checkerboard with superposed straight parallel lines (orange), indicates little pincushion or barrel distortion. Scale bar 10µm.

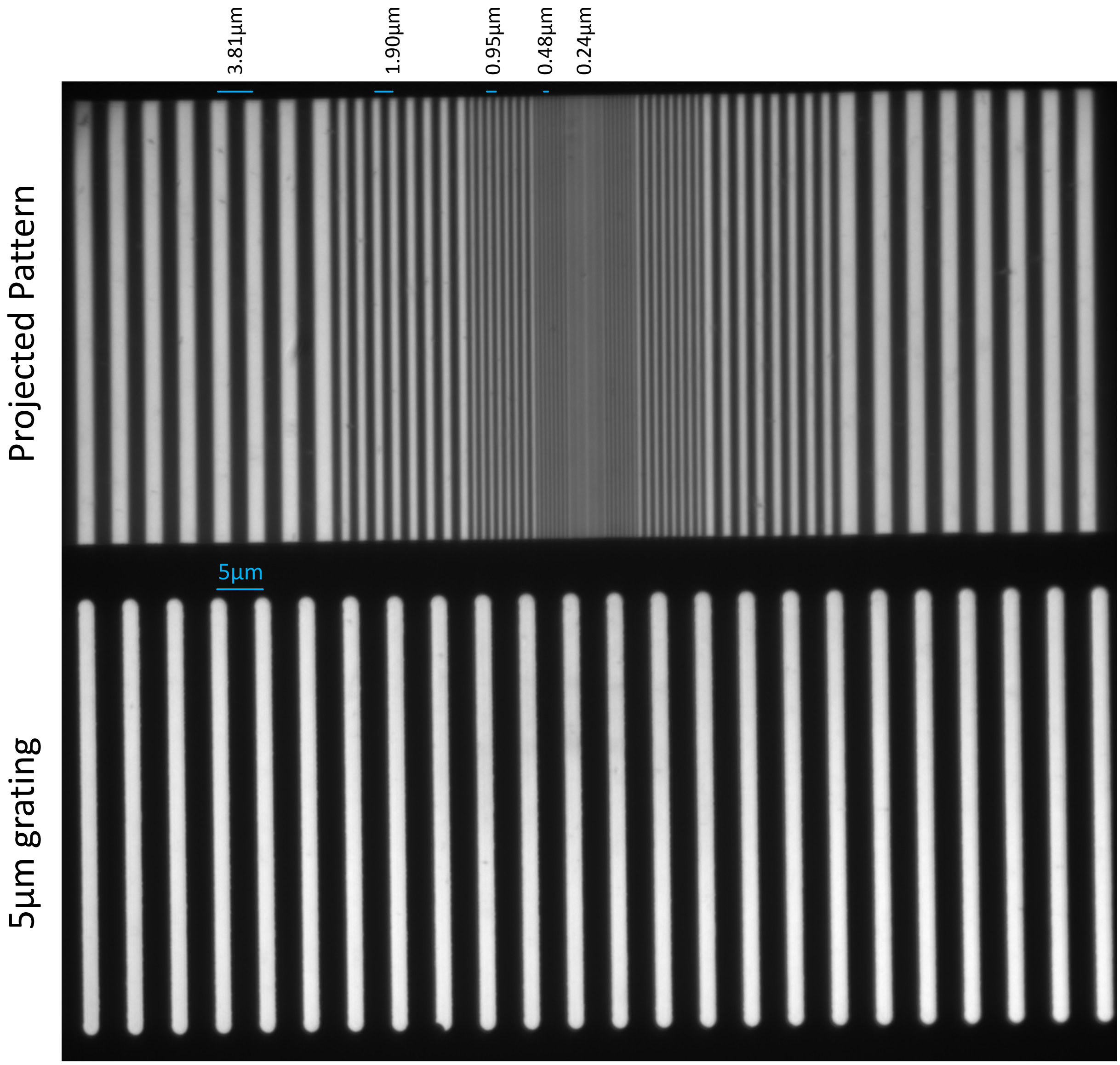

Supplementary Figure 8 | Resolution of projected images approaches diffraction limit Reflection image of a projected pattern of lines (top) above a 5µm-spaced calibration grating. The projected pattern of equal width dark and light (660nm illumination. Results are similar for 530nm) bands repeats from the edges to the center with periods of 3.81, 1.90, 0.95, 0.48, and 0.24µm. Images of projector and camera pixels at the sample focal plane are 0.12µm and 0.065µm on a side. Adjacent lines become unresolvable only at a spacing just above the optimal diffraction limit for this system (235nm, for NA=1.4, λ=660nm). Simultaneously resolved projected pattern and calibration grating indicate close alignment of camera and projector focal planes.

Experiment setup

- Initialize physical components.
- Initialize experiment parameters (locations, accessory files), data structure
- Choose software cell controllers. Initialize controller parameters.
- Location and cell-controller specification
    - Identify field location (x,y,) and focus (z).
    - Get offset of fluorescent image from focal plane
    - Get offset of projected patterns from focal plane. (1X)
    - Acquire, filter projector deshading images per stimulation channel (1X)
    - Calculate camera-projector homography (1X)
    - Identify local registration features.
    - Locate cell control regions for data extraction and stimulation, and assign controllers

Autonomous operation

- Loop (per time interval)
    - Update data and platform states (media).
    - Loop (per location)
        - Stage move. Focus to glass interface
        - Collect registration image. Calculate x,y offsets from location reference
        - Collect data images.
        - Apply fluorescence shading corrections, registration offsets
        - Extract cell data at control regions
        - Pass cell data to controllers.
        - Controllers verify cell validity and remove invalid cells (if required).
        - Controllers update internal states, return light control sequences per cell
        - Map light sequences to control image locations.
        - Apply inverse registration offsets, camera-projector homography, and projector shading corrections to control image,
        - Project control image. Deliver light stimulation to cells.

Supplementary Figure 9 | Feedback control experiment software function sequence.

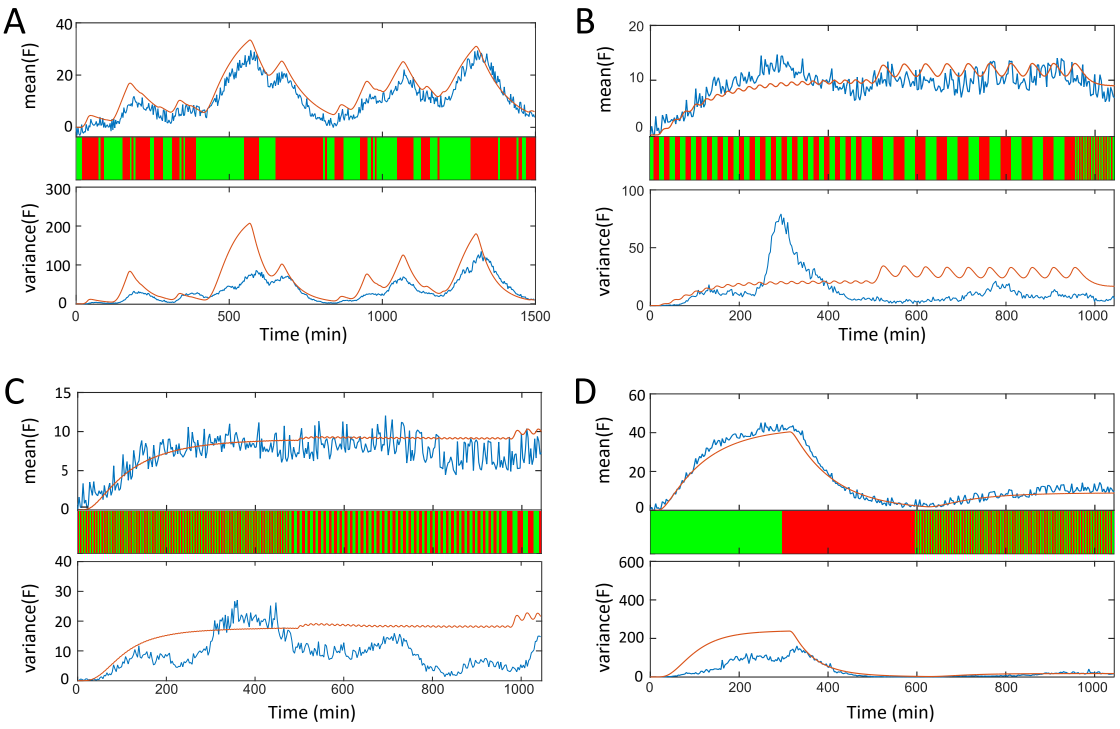

Supplementary Figure 10: **Inference data.** Model fits (red) and measured data (blue) used to calibrate the model. The applied light signals (same for all cells in each panel) are shown with red and green bars. (A) Measured mean and variance of 20 cells that were exposed to a random light sequence. (B) Measured mean and variance of 8 cells that were exposed to a light sequence alternating between green and red every 12min for the first 480min. Subsequently, we switched to a light sequence alternating between green and red every 24min and afterwards (at $t = 960$min) to a light sequence alternating between green and red every 3min. (C) Measured mean and variance of 7 cells that were exposed to a light sequence alternating between green and red every 3min for the first 480min. Subsequently, we switched to a light sequence alternating between green and red every 6min and afterwards (at $t = 960$min) to a light sequence alternating between green and red every 12min. (D) Measured mean and variance of 7 cells that were exposed to green light for the first 300min and then to red light for 300min. After that, the light was alternated between green and red every 3min.

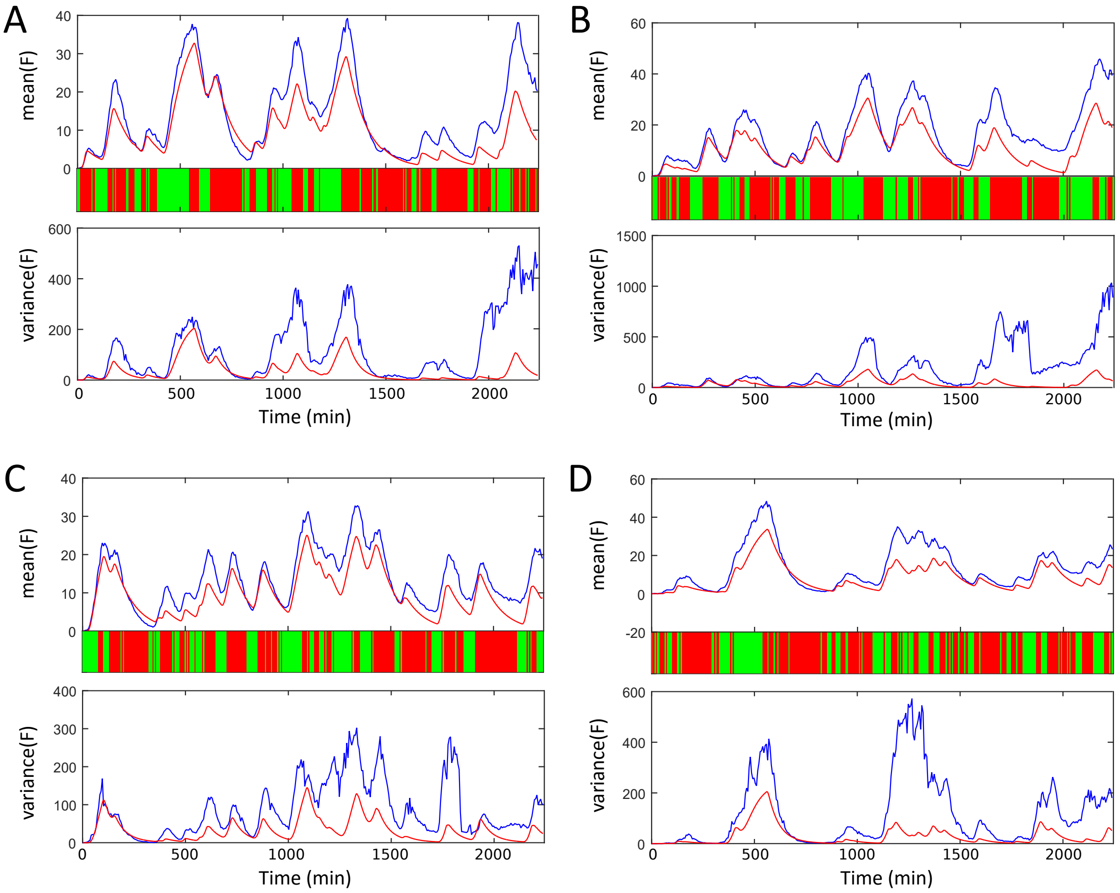

Supplementary Figure 11: **Validation data.** Model predictions (red) and measured data (blue) used to validate the model. Panels A-D show means and variances of 4 groups of cells that have been exposed to randomly chosen light sequences. The applied light signals (same for all cells in each group) are shown with red and green bars.

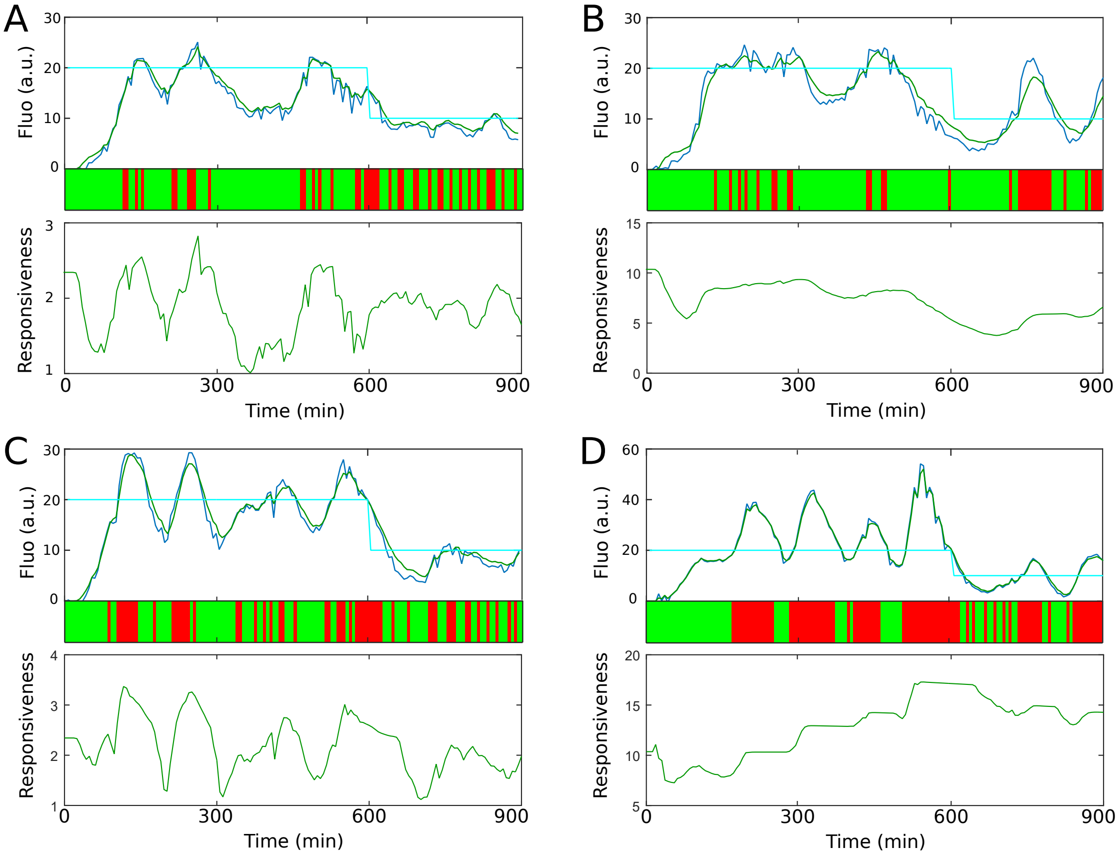

Supplementary Figure 12: **Comparison of the performance with different time scales of the cells' responsiveness.** (A,C) Cells controlled with large h_2. (B,D) Cells controlled with small h_2. In all panels, the blue lines correspond to measured fluorescence levels and the green lines to the estimated states that the filtering algorithm returned after processing the measurements. The applied light signals are shown below the fluorescence plots with red and green bars.

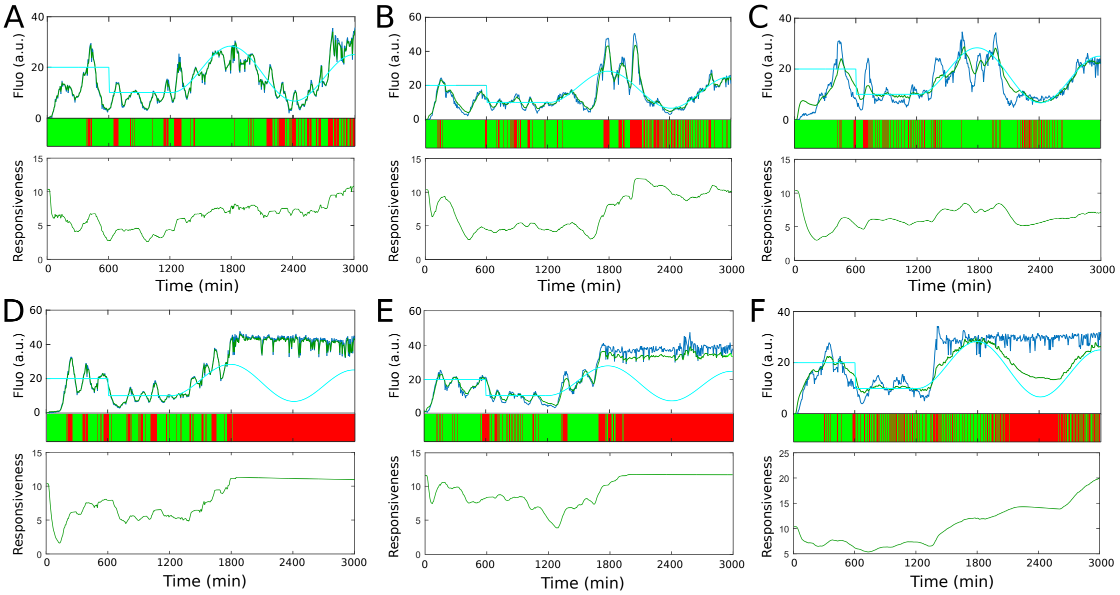

Supplementary Figure 13: **Comparison of the performance with different technical noise strength.** (A,D) With $R = 1$. (B,E) With $R = 10$. (C,F) With $R = 100$. In all panels we used the controller with $h_2 = 0.0004$. The blue lines correspond to measured fluorescence levels and the green lines to the estimated states that the filtering algorithm returned after processing the measurements. The applied light signals are shown below the fluorescence plots with red and green bars. Panels D to F show cells that halted growth after 1-2 days whereas panels A to C display cells that remained viable for more than 2 days.

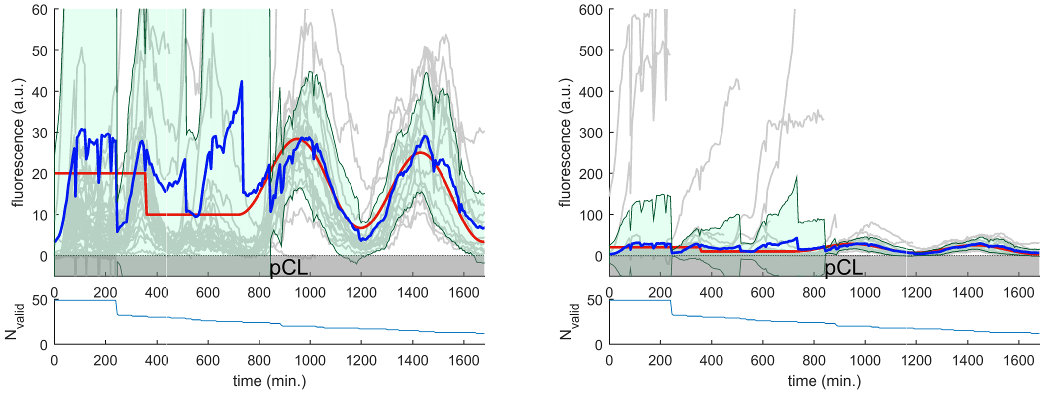

Supplementary Figure 14: **Population-level Closed Loop (pCL) control perturbed by outlier cells.** Feedback control of the population mean fluorescence using the same model predictive control algorithm as used for iCL control. The mean fluorescence of the population is calculated by averaging the fluorescence levels of the normally growing cells. Invalidation of pathological cells is automatically performed using a real-time implementation of the algorithm described in the Methods Section of the main paper to classify cells. As soon as a cell is classified as pathological, it is removed from the analysis and does not contribute to the population mean anymore. Since some pathological cells are only detected with a delay of several hours and often show extremely large fluorescence levels before they are removed, the calculated mean fluorescence is significantly affected and the controller compensates for the outlier cells by applying less green light to the population. As a consequence, the fluorescence of almost all normally growing cells is significantly below the target whenever outlier cells are present. At the time points when the outlier cells are removed from the analysis the mean fluorescence displays large jumps. Individual cell trajectories are shown by the grey lines up to the time point when the corresponding cell was removed from the analysis. The two panels display the same data with different scaling of the y-axis. The number of cells classified as growing normally is shown at the bottom. Removal of pathological cells starts only after 240min have passed and sufficient data for the classification has been collected.

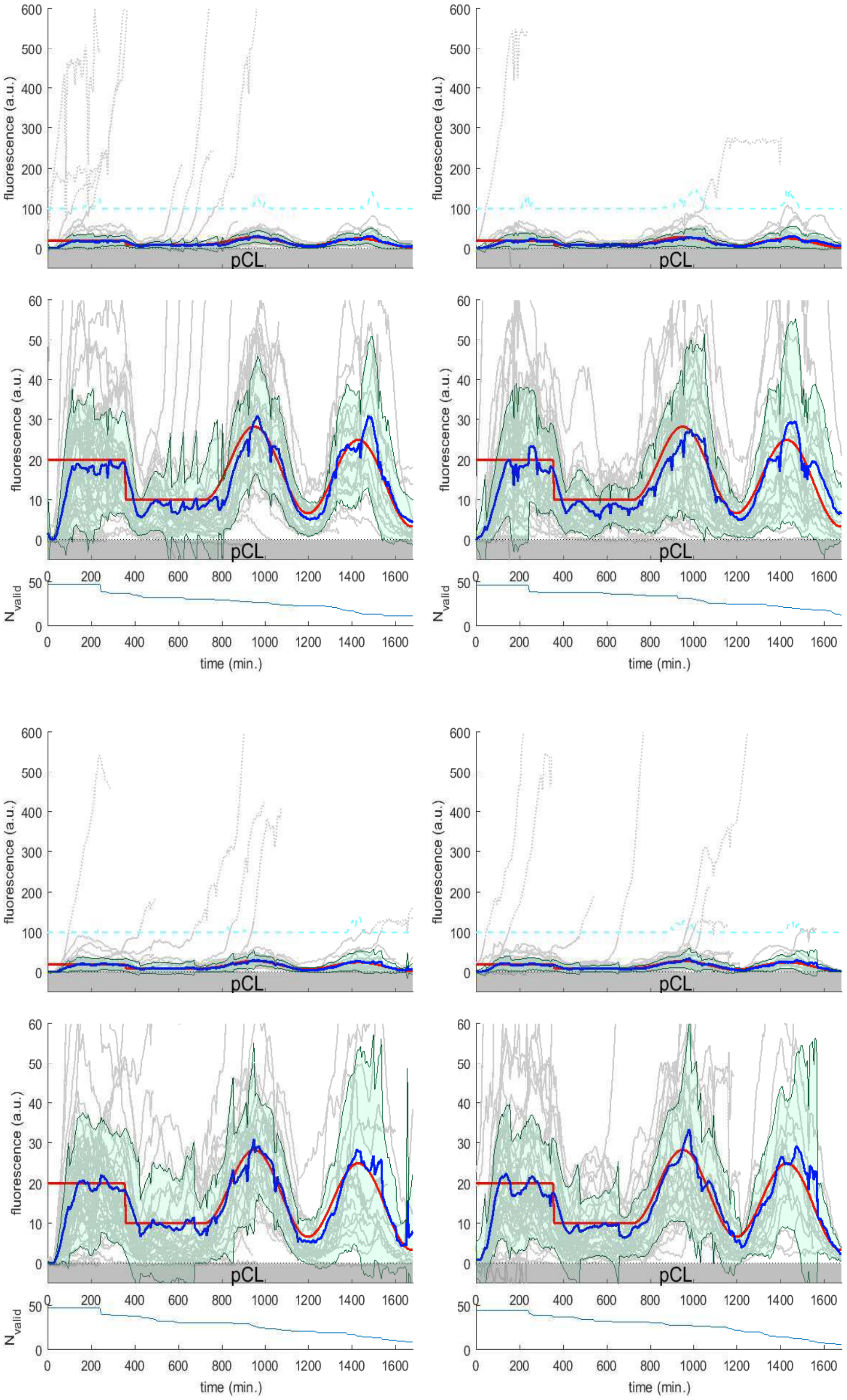

Supplementary Figure 15: (Continued on the following page)

Supplementary Figure 15: **Population-level Closed Loop (pCL) control with improved classification of cells.** The mean fluorescence (blue) of four groups of 50 cells (all in a single experiment) is controlled using pCL to follow a pre-defined target profile (red). Population means are calculated after filtering out pathological cells in real time using our cell classification algorithm as well as removing any cell whose fluorescence exceeds 100 a.u. and 10 median absolute deviations from the population median. Top and bottom panels show the same data with different scaling of the y-axis. Individual cell trajectories are displayed with grey lines. Trajectories that end during the experiment correspond to cells that were classified as pathological. Trajectories that turn from solid to dotted correspond to cells that were classified as pathological only because they exceeded the threshold (cyan) in the controlled fluorescence channel. All dotted lines eventually end implying, on the one hand, that we did not remove any normally growing cells, and on the other hand, that the detection of pathological cells without using a threshold on the controlled fluorescence channel can be successful but only with a potentially large delay.

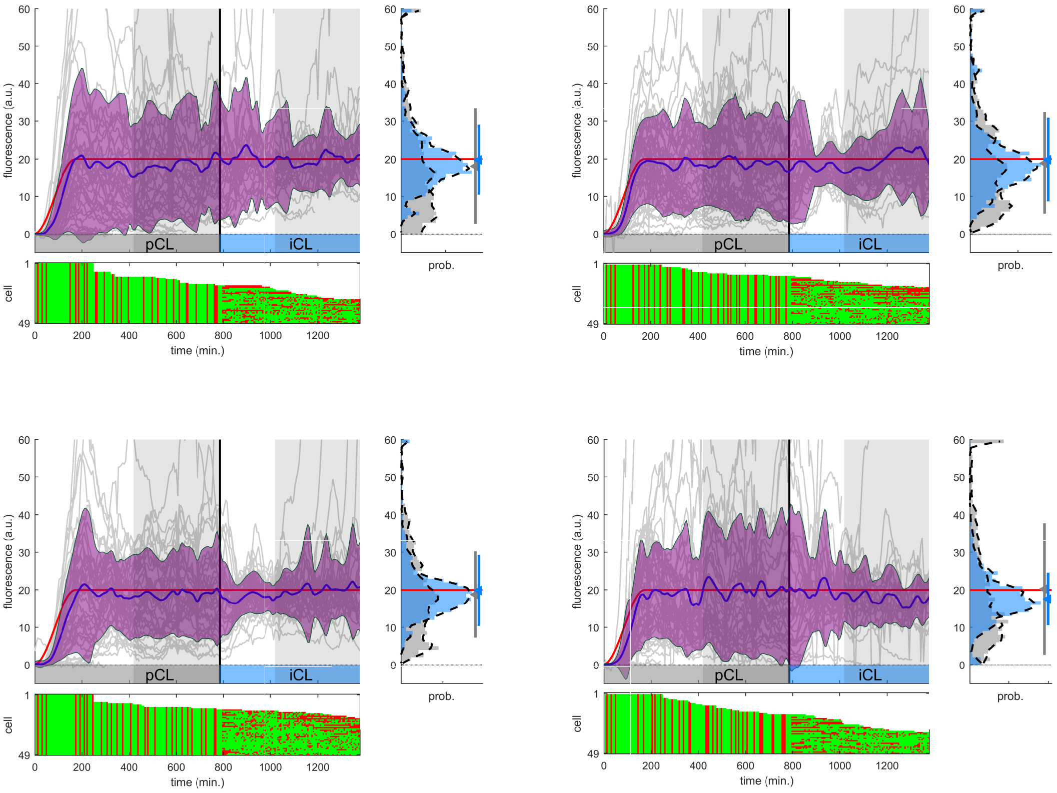

Supplementary Figure 16: **Switch from pCL to iCL control.** Four groups of 49 cells (all in a single experiment) with 20 a.u. as constant control target. For the first 800 minutes the mean fluorescence was controlled using pCL with global light stimulation applied to the entire population. The mean fluorescence was calculated using the improved real-time classification of the population into normally growing and pathological cells (as in Supplementary Figure 15). After 800 minutes control was switched to iCL mode such that cells received individualized light signals. The light sequences applied to all cells are displayed at the bottom of each panel. Whenever a light sequence ends, the corresponding cell was classified as pathological and removed from the analysis. Histograms at the right of each panel display and compare the typical spread of fluorescence values around the target profile in the two control modes. These histograms have been calculated based on all measured fluorescence values of all normally growing cells during the grey shaded time intervals. The displayed spreads around the target for iCL control tend to be larger here than Figure 3a,b in the main paper because the calculation of population statistics has been done based on real-time classification of cells to obtain results comparable to pCL control.

Supplementary Table 1: **Inferred parameter values.** c_2, a, b, and h_2 are in 1/min, τ_{delay} is in min, $\mu_{\text{E}}, n_{\text{H}}$ and κ are unitless, and s is in arbitrary units.

c_2	a	b	s	μ_{E}	h_2	n_{H}	κ	τ_{delay}
0.0631	0.2827	0.0104	0.9958	2.3435	0.0303	3.6655	0.4851	12

Supplementary Methods

Modeling of the system.

Description of the model.

In this section, we describe the model used by the controller in the main paper. Above all else, there are three main requirements on the model:

(i) **Predictiveness:** The model needs to be sufficiently predictive over the planning horizon h such that it can inform the controller about the expected consequences of different decisions.

(ii) **Adaptability:** In order to cope with the large amount of cell-to-cell variability, predictiveness for each individual cell can only be achieved if the model can adapt itself to attributes of particular cells. The model needs to know that there is variability between cells, it needs to be able to online learn the attributes of each cell from the collected data, and it also needs to be aware that cells can change over the course of the experiment.

(iii) **Efficiency:** We desire to control hundreds of cells simultaneously and in our experimental setting new measurements need to be processed and new decisions need to be made every six minutes. To reach the best decisions, the controller needs to search the set of possible light sequences over the planning horizon and determine the expected fluorescence values for every cell and every light sequence. It is therefore a prime prerequisite that the model can be evaluated very quickly and on the lab-computer that is controlling the experiment.

In the light of these requirements, we aimed to construct the simplest model that fulfills (i) and (ii) and chose to represent the system with three state variables: one variable (H) to capture the dynamics of the light-activation system, one variable (E) that lumps together all sources of cell-to-cell variability, and one variable (F) that represents the fluorescence in the cells and corresponds to the measurable output of the system. To keep the model easily analyzable while still capturing the key features of the system, we decided to assume that the light-activation dynamics are deterministic and the same in all cells and represented them with a linear differential equation with an input delay:

$$\frac{\partial}{\partial t} H(t) = u(t - \tau_{\text{delay}}) - c_2 \cdot H(t), \tag{1}$$

where $u(t) = 1$ if the light signal applied to the cell at the last intervention time point before time t has been green and $u(t) = 0$ if it has been red. Note that the light signal is not evaluated at the current time t but instead at $t - \tau_{\text{delay}}$, i.e. we assume that there is a delay of τ_{delay} minutes in the light activation system and only after this delay $H(t)$ starts to increase. An equivalent delay would also be obtained with a fluorescence reporter that has a non-zero deterministic maturation time. Hence, the parameter τ_{delay} can be thought of as capturing both delays in the light system as well as in the reporter system. The rate at which proteins are produced in the cells is then assumed to depend non-linearly (but deterministically) on the state of the light system according to a Hill function that converts $H(t)$ to the a new variable $L(t)$. To capture cellular individuality, the remaining part of the model (time varying cell-to-cell variability, protein production and dilution) is represented as a stochastic (but linear) reaction network:

$$\emptyset \xrightarrow{\quad h_1 \quad} E \xrightarrow{\quad h_2 \quad} \emptyset, \qquad\qquad \emptyset \xrightarrow{\quad a \cdot E(t) \cdot L(t) \quad} F \xrightarrow{\quad b \quad} \emptyset,$$

$$\text{where} \quad L(t) = \frac{(H(t) \cdot c_2)^{n_{\text{H}}}}{\kappa + (H(t) \cdot c_2)^{n_{\text{H}}}}. \tag{2}$$

In particular, we decided to lump all sources of extrinsic variability (i.e. numbers of ribosomes, plasmid copy numbers, etc.) together and to describe them with the one-dimensional stochastic process $E(t)$, which multiplies (and thus individualizes) the cells' fluorescent protein production rates. $E(t)$ is interpreted as the randomly fluctuating cell responsiveness to green light throughout the manuscript. We assume that $E(0) \sim Poisson(\frac{h_1}{h_2})$, which implies that $E(t)$ is a stationary process and can also be characterized by the mean (or variance) of the initial distribution $\mu_{\text{E}} := \mathbb{E}[E(0)] = Var(E(0)) = \frac{h_1}{h_2}$ and its time scale h_2. Note that the Poissonian nature of this process implies that population mean and variance of this noise process are always the same. However, $E(t)$ enters the downstream model only after multiplication with the additional free parameter a, which decouples mean and variance and enables the model to also represent more general scenarios. To keep the model simple, we did not incorporate promoter switching or mRNA dynamics and represented only the fluorescent protein as a stochastic birth-death process in which the product $E(t) \cdot L(t)$ of a cell's responsiveness and the state of its light system is directly proportional to the rate of birth events, i.e. to the production of fluorescent proteins in the cell. The frequency of death events is determined by the parameter b and corresponds to degradation/dilution. Finally, we assume that each fluorescent protein emits a deterministic amount of fluorescence, i.e. that the measured fluorescence signal is directly proportional to the number of fluorescent protein molecules with proportionality constant s. To summarize, the model comprises the 9 parameters $\theta = \{c_2, a, b, s, \mu_{\text{E}}, h_2, n_{\text{H}}, \kappa, \tau_{\text{delay}}\}$.

Due to the linearity of the stochastic part of the model, it is possible to efficiently and exactly propagate uncertainty through the dynamics [1]. In particular, equations for the time evolution of the moments of the system can readily be derived from the underlying master equation [2, 3, 4]. Up to second order moments, the full system's dynamics are

given by:

$$\frac{\partial}{\partial t}H(t) = u(t-\tau_{\text{delay}}) - c_2 \cdot H(t), \qquad L(t) = \frac{(H(t)\cdot c_2)^{n_{\text{H}}}}{\kappa + (H(t)\cdot c_2)^{n_{\text{H}}}}, \tag{3}$$

$$\frac{\partial}{\partial t}\mathbb{E}\left[E(t)\right] = h_1 - h_2 \cdot \mathbb{E}\left[E(t)\right] \tag{4}$$

$$\frac{\partial}{\partial t}\mathbb{E}\left[F(t)\right] = a \cdot L(t) \cdot \mathbb{E}\left[E(t)\right] - b \cdot \mathbb{E}\left[F(t)\right] \tag{5}$$

$$\frac{\partial}{\partial t}\mathbb{E}\left[E(t)^2\right] = h_1 + 2h_1 \cdot \mathbb{E}\left[E(t)\right] + h_2 \cdot \mathbb{E}\left[E(t)\right] - 2h_2 \cdot \mathbb{E}\left[E(t)^2\right] \tag{6}$$

$$\frac{\partial}{\partial t}\mathbb{E}\left[E(t)F(t)\right] = h_1 \cdot \mathbb{E}\left[F(t)\right] + a \cdot L(t) \cdot \mathbb{E}\left[E(t)^2\right] - (h_2 + b) \cdot \mathbb{E}\left[E(t)F(t)\right] \tag{7}$$

$$\frac{\partial}{\partial t}\mathbb{E}\left[F(t)^2\right] = b \cdot \mathbb{E}\left[F(t)\right] + a \cdot L(t) \cdot \mathbb{E}\left[E(t)\right] + 2a \cdot L(t) \cdot \mathbb{E}\left[E(t)F(t)\right] - 2b \cdot \mathbb{E}\left[F(t)^2\right] \tag{8}$$

Inference of the model parameters.

To determine appropriate model parameters we performed two calibration experiments in which we exposed the cells to different light sequences. In the first experiment, all cells were exposed to an identical random light sequence that switched between red and green with squared, Poisson-distributed waiting times (Supplementary Figure 10A). The second experiment was geared towards characterizing the dynamics of the light activation system. To this end, we split the cells into three groups and exposed each of the groups to light sequences with periodic switching between green and red light at different frequencies. Means and variances of the dynamics of the measured responses for the different groups are shown in Supplementary Figure 10. The complicated dynamics of the light activation system can immediately be seen from this data because the cells respond very differently to the different light sequences, despite the fact that averaged over time all cells received exactly the same amount of green and red light.

To infer the model parameters from this data we calculated means and variances over the fluorescence values of all cells at all the measurement time points (i.e. every six minutes). We then made use of the Bayesian moment-based inference scheme proposed in [5] with the same Metropolis-Hastings Markov chain Monte Carlo algorithm to search the parameter space for the values with maximum a posteriori probability (MAP estimates). Slightly differently to [5], however, we did not estimate the variance of the data from the data itself, but instead calculated the likelihood entirely from the model according to the formulae provided in [6, 7]. This requires iteratively evaluating moment equations of the stochastic model up to fourth order (these equation can easily be automatically derived with the code that we published in [3]), but the computational expense is bearable for our model - in particular, because parameter inference is an offline calculation that can be performed without time constraints imposed by the real system. To ensure that good parameters are found by the algorithm, we multi-started it from different locations in parameter space and extracted the parameter values with the highest maximum a

posteriori probability of all the runs. The results are given in Supplementary Table 1. This analysis also showed that some of the model parameters are not very well identifiable from means and variances of the data. However, this is not a particularly relevant concern for our study as long as the model with the inferred parameters is predictive in the regime in which we are operating the system. One thing that has to be pointed out, however, is that the parameter h_2 describing the time scale of the extrinsic fluctuations is only very weakly identifiable. This is because the information about this time scale is encoded primarily in individual trajectories and cannot be recovered very well from means and variances of the population. Consequently, a parameter inference scheme that operates on single cell trajectories would be necessary to assign a reliable data-inferred number to h_2 [8]. We decided against using such an inference scheme, however, and instead chose to not assign a directly data-related meaning to the parameter, but to treat it as a free tuning parameter of the controller. In fact, this parameter is of key importance for determining how noise is filtered in the control scheme because it determines how quickly the controller forgets and readjusts the responsiveness of the cell that it is trying to control. A more in-depth discussion and a study of the performance of the controller with different values of h_2 will follow in the next section. Finally, it should be noted that the parameter τ_{delay} was originally identified as 11.7791min and subsequently rounded to 12min. The corresponding difference of less than 15 seconds is practically irrelevant but makes the control algorithm simpler (and thus faster) because sent light signals start to affect the system exactly two 6min-cycles after they have been applied to the cells.

Finally, note that the experiments in Supplementary Figure 10 used a motile E.coli strain (CR138, Methods), and subsequent experiments were performed with a non-motile variant. In particular, for each of the light sequences in Supplementary Figure 10 only a handful of cells provided valid signals over the entire experiment (implying large uncertainties specifically for the measured variances in Supplementary Figure 10). This motivated us to switch to a flagellin-deleted, non-motile strain (CR145, Methods) to increase the number of cells that remain within the detection regions throughout the entire experiment. Furthermore, the data also showed high frequency oscillations that were approximately the same for all cells and independent of the applied light sequence (clearly visible in the shown means and variances in Supplementary Figure 10, in particular in the mean in panel C). We were able to attribute these oscillations to a slow, additive fluctuation in the camera signal (apparent even in unexposed images), and could remove much of it by subtracting the median of 10 dark frames collected immediately prior to the data image.

To test the updated experimental setup, we applied four different randomly chosen light sequences to four groups of cells. Other than the removed high frequency oscillations and a larger number of valid cells per light sequence, there was hardly any noticeable difference to the previous data. In fact, somewhat surprisingly, the model identified from the data in Supplementary Figure 10 predicted the means and also the variances of the cells' responses quite well for all four light sequences (see Supplementary Figure 11). We concluded that re-identification of the model parameters was not necessary and kept all model parameters as displayed in Supplementary Table 1.

Model predictive control.

A high level picture of the inner workings of the controller has been provided in the main paper (Figure 2). In this section, we provide more details and state the precise mathematical concepts and equations that are used by the controller. In general, the used control scheme is based on ideas of model predictive control with a model to predict the future time-evolution of the system, a state estimator to infer the complete state of the system from the measured output and a finite receding planning horizon. We chose to operate the controller on 6min cycles, that is every 6 minutes the cells' fluorescence is recorded and new light signals are sent to the cells. In principle, our experimental platform can also be operated with shorter cycles. However, given the slow time scale of gene expression it is unlikely that a significant gain in control performance can be obtained by measuring fluorescence and adjusting the light inputs more frequently than every 6 minutes. On the contrary, 6 minute intervals allow more time for the calculation of the best light signals and enable us to control a larger number of cells in parallel and/or to run the receding horizon control algorithm with a longer planning horizon.

As a further note, our current implementation of the control algorithm chooses the next light signals by predicting the expected response for all cells and all possible light sequences over the planning horizon. Since the number of possible light sequences grows exponentially in the planning horizon, the computational cost of the implemented algorithm scales linearly in the number of cells and exponentially in the planning horizon. If computation time starts to be the limiting factor, e.g. because more cells are to be controlled with longer planning horizons, it will become necessary to speed up this calculation, e.g. by choosing only out of a set of representative light sequences or by implementing optimization algorithms that aim to find the best light sequence without testing all possibilities. For the results presented in this paper, however, this was not necessary.

State estimation.

The task of the state estimator is to separate technical measurement noise from fluorescence fluctuations that are caused by noise and variability in gene expression in the cell. In other words, it has to read the measured fluorescence values that it is receiving from the microscope and process these values into an estimate of the full state of the system, i.e. into the actual true level of fluorescence in the cell (variable F in the model) and the cell's responsiveness (variable E in the model). This task requires noise models for the system and the technical noise and is the reason why a stochastic description of the model was derived. For technical noise, we assumed additive and time invariant Gaussian noise with mean zero and variance R. This noise variance determines how large unexpected fluctuations in the measured fluorescence levels must be in order to be attributed to real changes in the cell's responsiveness instead of being rejected as technical noise. In our implementation, we tuned R to achieve the desired noise rejection properties of the filter (see Supplementary Figure 13). Alternatively, one could also attempt to infer R from measured data. With this parameter specified, the state estimation is performed based

on the ideas of hybrid Kalman filtering and described below. In the following, we will denote by u_k the light signal sent to the cell at time $k \cdot 6$min and set $d = \frac{\tau_{\text{delay}}}{6}$.

Initialize: At time $t = 0$, set $k = 0$, initialize $u_{-2} = u_{-1} = 0$ (corresponding to red light), and set u_0 to the light applied at $t = 0$ and the state estimate $\hat{\mathbf{x}}_{0|0}$ to

$$\hat{\mathbf{x}}_{0|0} := \begin{bmatrix} \mathbb{E}[E(0)] \\ \mathbb{E}[F(0)] \end{bmatrix} = \begin{bmatrix} \mu_{\text{E}} \\ 0 \end{bmatrix},$$

where the first component of the vector corresponds to the best estimate of the cell's responsiveness (without any information from the particular cell this is the mean inferred in the calibration experiments), and the second to the cell not having any fluorescent protein. Furthermore, set the covariance matrix $\mathbf{P}_{0|0}$ of $\hat{\mathbf{x}}_{0|0}$ to

$$\mathbf{P}_{0|0} := \begin{bmatrix} Var(E(0)) & Cov(E(0), F(0)) \\ Cov(E(0), F(0)) & Var(F(0)) \end{bmatrix} = \begin{bmatrix} \mu_{\text{E}} & 0 \\ 0 & 0 \end{bmatrix}.$$

Note that μ_{E} in this equation represents the uncertainty (i.e. variance) of the cell's responsiveness and is the same as the best estimate of the cell's responsiveness because E is assumed to have a Poisson distribution over the population.

Iterate from $k = 1$ to $k = k_{\text{end}}$:

1. At time $t = k \cdot 6$min receive a new incoming fluorescence measurement y_k from the microscope.
2. Calculate the best a priori (i.e. without incorporating y_k) state estimate at the next measurement time $\hat{\mathbf{x}}_{k|k-1} = f(\hat{\mathbf{x}}_{k-1|k-1}, u_{k-1-d})$ by propagating $\hat{\mathbf{x}}_{k-1|k-1}$ through the dynamics f of the system's means (Eq. 3-5).
3. Calculate the covariance matrix $\mathbf{P}_{k|k-1} = g(\hat{\mathbf{x}}_{k-1|k-1}, \mathbf{P}_{k-1|k-1}, u_{k-1-d})$ of $\hat{\mathbf{x}}_{k|k-1}$ from the covariance matrix $\mathbf{P}_{k-1|k-1}$ by making use of the second order moment equations g (Eq. 3-8).
4. Calculate the a posteriori (after incorporating y_k) state estimate and its covariance matrix:

$$\begin{aligned} &\hat{\mathbf{x}}_{k|k} = \hat{\mathbf{x}}_{k|k-1} + \mathbf{K}_k \cdot (y_k - \mathbf{C} \cdot \hat{\mathbf{x}}_{k|k-1}), \\ &\mathbf{P}_{k|k} = (\mathbf{I} - \mathbf{K}_k \cdot \mathbf{C}) \cdot \mathbf{P}_{k|k-1}, \text{ where} \\ &\mathbf{C} = [0 \ s] \text{ and } \mathbf{K}_k = \mathbf{P}_{k|k-1} \cdot \mathbf{C}^\top \cdot \left(\mathbf{C} \cdot \mathbf{P}_{k|k-1} \cdot \mathbf{C}^\top + R\right)^{-1}. \end{aligned}$$

From the theory of Kalman filtering, it is known that this state estimation leads to minimal mean squared error estimates $\hat{\mathbf{x}}_{k|k}$ if both $\hat{\mathbf{x}}_{k|k-1}$ and y_k have a Gaussian distribution. In our case, the distribution of $\hat{\mathbf{x}}_{k|k-1}$ is not exactly Gaussian and hence

the resulting state estimates are not guaranteed to be optimal. We nevertheless rely on this scheme because it only requires up to second order moments and allows us to avoid the difficult and computationally too expensive calculation of the full distribution of $\hat{\mathbf{x}}_{k|k-1}$. A more in-depth discussion of approximate state estimation techniques for biochemical reaction networks can be found in reference [9].

Calculation of the optimal light sequence.

At each iteration, the obtained state estimate $\hat{\mathbf{x}}_{k|k}$ is passed on to a second algorithm whose goal it is to choose the best light input u_{k+1} for the next intervention time point. First, the state $\hat{\mathbf{x}}_{k+1|k}$ at the next intervention time point has to be predicted because it is still unknown but cannot be influenced anymore by the controller. Due to the delay of $\tau_{\text{delay}} = 12$min, i.e. $d = 2$ intervention cycles, the same holds for the state of the system in the 12min following the next intervention time point. Accordingly, the controller uses the model and the past light signals u_{k-2}, u_{k-1}, u_k to predict the state $\hat{\mathbf{x}}_{k+1+2|k}$ that the system is expected to be in 18min after the collection of the last fluorescence measurement. From that time point on, the system's state starts to depend on light signals that have not been applied yet and can therefore be influenced. Note, however, that due to the slow dynamics of the light system and maturation of the reporter it takes significantly more than 18min until a clear fluorescence response is detectable.

An important feature of the controller is that it is operating in a receding horizon fashion with a planning horizon that we chose as 48min, i.e. $h = 8$ intervention cycles. This means that at each iteration the controller searches through the set of all light sequences $u_{k+1}, \ldots, u_{k+1+h}$ despite the fact that it is only required to make a decision on u_{k+1}. This is crucial because the system responds very slowly to the applied light inputs and the best decision that would be reached for u_{k+1} without a planning horizon might not be a good long-term strategy. In particular, if the target fluorescence trajectory is set to be time-varying (as in almost all of our experiments), it is important that the controller anticipates upcoming changes in the target and adjusts the light inputs that it sends to the cells sufficiently early. The optimization problem that is solved by the controller for each cell to determine u_{k+1} is therefore the following:

$$\min_{u_{k+1},\ldots,u_{k+1+h}} \sum_{s=k+2+d}^{k+2+d+h} \left(\mathbf{C} \cdot \hat{\mathbf{x}}_{s|k} - Target(s)\right)^2, \tag{9}$$

where we decided to minimize the sum of squares of the predicted deviations from the target over the planning horizon, i.e. to weight all future time points equally.

Parametrization of the control and filtering algorithms.

As stated in the previous sections, we treated the time scale of fluctuations in the cells' responsiveness h_2 and the variance of the technical noise R as tuning parameters of the control algorithm. In principle, these parameters are inferable from experimental data but given that we used a highly simplified model in our study it is questionable whether

meaningful values would really be learned in this way. For instance, we observed that cells sometimes display very quick and large changes in their responsiveness but at other times remain approximately equally responsive over very long time periods - a feature that could not be captured by the simple one-state model with a single time scale h_2 for the cells' responsiveness. Similarly, given that the model is only a crude simplification of the real biological process, it is more likely that an inferred value for R would mostly represent model mismatch instead of technical measurement noise. Since both these parameters are of key importance for the performance of the controller, we decided to treat them as tuning parameters of the control algorithm and performed a dedicated experiment to test the performance with different values.

It should be pointed out that, while h_2 and R can be adjusted independently for each controller, they are not changed during the experiment and need to be pre-specified before the experiment is started. Accordingly, controlling different cells with different values for these parameters can serve to test different parametrizations of the control algorithm in parallel but not to capture and compensate differences between the controlled cells. However, one could envision to extend the current approach and to estimate cell specific values for the parameter h_2 from incoming data during the experiment in the same way as the cells' responsiveness is estimated in our current implementation. Such an extension would correspond to the assumption that not only the responsiveness might be different in different cells, but also the time scale on which this responsiveness is fluctuating might vary between cells and possibly also in time.

Supplementary Figure 12 and Supplementary Figure 13 show a closed loop control experiment where different groups of cells were controlled with different settings of the controller. In particular, Supplementary Figure 12 provides a comparison between a setting with the inferred value $h_2 = 0.0303$, which corresponds to rather fast fluctuations in the cells' responsiveness, and a setting with $h_2 = 0.0004$ corresponding to much slower fluctuations [1]. To illustrate the difference between the two controller settings, we chose to display four cells of that experiment. The cells in panel A and C have been controlled with a fast time scale of the cells' responsiveness. Panel A shows a cell that displayed several quick changes in its responsiveness, but given that the controller expected fast fluctuations it was still capable of controlling the cell's fluorescence decently well. Panel C, on the other hand, shows a cell where the fast updates of the cell's responsiveness made by the controller were disadvantageous. In particular, the controller first learned that this cell is very responsive (around $t = 100$ minutes), forgot this again in the following period that had little green light (around $t = 100$ to $t = 200$), only to be surprised that the cell was still very responsive after $t = 200$ minutes. The cells in panel B and D have been controlled with a slow time scale of the cells' responsiveness. Panel B shows a cell that displayed almost no changes in its responsiveness and is therefore well controlled. The cell in panel D, on the other hand, showed a large increase in its responsiveness at around $t = 200$min (visible in the sudden overshoot of the fluorescence level over the target shortly after $t = 200$min). However, due to the small value of h_2 in

[1] Note that the parameter h_2 is not structurally unidentifiable, which implies that changing h_2 requires one to also readjust all the remaining model parameters.

the model, the controller judged such a large change to be implausible and only updated its belief about the cell's responsiveness by a moderate amount. At around $t = 280$min, when the next green light needs to be applied to the cell, it becomes visible that this update was not sufficient and that the controller underestimated the true responsiveness of this cell. The consequence is that it applied too much green light leading to a second overshoot of the fluorescence level over the target.

For the results in the main paper, we eventually decided to use the controller with $h_2 = 0.0303$. We would like to point out, however, that an important strength of our experimental platform is that algorithms can be assigned on a cell by cell basis, i.e. it is perfectly possible and even straightforward to run hundreds of different control algorithms in a single experiment. In fact, we made use of this here and tested the performance for different values of R within the same experiment. Supplementary Figure 13, panels A-C, show cells that have been controlled with $h_2 = 0.0004$ and $R = 1, 10, 100$. It can be seen that the larger R is, the larger are the deviations of estimated fluorescence (green) and measured fluorescence (blue) because larger parts of unexpected changes are attributed to technical measurement noise. Very illustrative is also the behavior of the filtering algorithm for cells that halted growth, thereafter maintaining constant fluorescence levels (panels D-F). For $R = 1$ the algorithm completely believes the measurements and ignores that this is totally implausible according to the model. For $R = 10$ it corrects the values to some degree based on what the response should be according to the model. For $R = 100$ it almost completely labels these values as technical noise and thinks that the true fluorescence is still being controlled. Based on these results, we judged $R = 10$ to be the most plausible and implemented this value for the results in the main paper.

Comparison of individual-level (iCL) and population-level (pCL) control.

In the main paper, we provided a dedicated comparison of open loop control (OL) and individual-level closed loop control (iCL) but did not perform any population-level closed loop control (pCL) experiments. pCL has been the state of the art for population control in the past and it is therefore of interest to evaluate our iCL results by comparing them directly to results obtained with pCL control. In the past, pCL control experiments have been performed in flasks and the mean fluorescence of the population has been measured by taking samples from the flask and controlled by exposing the entire population to light [10]. An important feature in this is that the population that is being measured and controlled is constantly growing. As a consequence, dead cells or cells that have halted growth do not accumulate throughout the experiment, remain always only a minor fraction of the population, and presumably have only a negligible influence on the measured population mean fluorescence. Contrary to this, in experiments performed in a mother machine, all newly born daughter cells are pushed out of the channels and leave the chip such that the number of normally growing cells is not increasing over time. It follows that pathological cells (e.g., dead or non-growing cells or cells that have lost one or both light system plasmids) are not diluted but instead accumulate throughout the experiment. Mean fluorescence and behavior of a population of cells in a mother machine device can thus diverge enormously over time from the mean fluorescence and

behavior of cells that are periodically sampled from a bulk culture. To nevertheless be able to compare iCL to control results obtained in bulk cultures, we developed a cell classification algorithm that extracts the normally growing cells from all cells in the mother machine device. To avoid invalidating cells due to normal variation, we kept the classification of cells as orthogonal as possible to the control circuit and only invalidated cells based on the recorded growth data, the expression levels of the constitutive reporter gene, noisiness of fluorescence measurements, quality of initialization (erroneously high background fluorescence levels at time zero and early trajectories that trend significantly negative), and if a long moving average of the estimated cell responsiveness drops below a threshold (see the Methods Section in the main text). Population mean fluorescence and variance around the mean are then calculated only after all pathological cells have been filtered out.

It is important to note, however, that the cell classification algorithm requires information about growth and expression of the constitutive reporter collected over the entire experiment to reliably classify cells. Stated differently, it performs an offline calculation after the experiment is finished and cannot directly be used online for real-time classification of cells since it would require information that has not yet been recorded. While this is sufficient for calculating fluorescence means and standard deviations of iCL controlled cells at the end of the experiment, it does not allow us to quantify the behavior of the normally growing population in real time, which would be desirable as it would allow us to perform pCL control on our platform and to compare the results to our iCL results. Thus, as a next step, we tested how reliably the cell classification algorithm could operate in real time to detect and remove pathological cells based only on the data that has been observed up to the current time. The results showed that, while all pathological cells were eventually successfully filtered out, some cells only became distinguishable from the normally growing cells several hours after they started to show abnormal (typically extremely large) fluorescence signals for the light induced gene. As a consequence, calculation of the population mean fluorescence is significantly affected by these cells and pCL feedback control of the mean fluorescence did not provide satisfactory results (see Supplementary Figure 14). [2]

To obtain improved performance of pCL control, we adjusted the cell classification algorithm to also exclude any cell whose fluorescence level becomes larger than a threshold of 100 a.u. and 10 median absolute deviations from the population median. These fluorescence levels cannot be reached by normally growing cells even if they are exposed to full green light. pCL control results using the updated cell classification algorithm in real time are shown in Supplementary Figure 15. It can be seen that control of the mean is significantly improved. Comparing these results to the control results in Figure 3a in the main paper, we can conclude that pCL performs similarly as iCL for controlling the

[2] It should be pointed out that the degree to which the control performance is affected by outlier cells depends on the population feature that is being controlled. For instance, if we had attempted to control the median fluorescence instead of the mean, the performance of the algorithm would most likely have been satisfactory. We decided to not use the median because the mathematical basis of our MPC controller has been established for the mean (e.g. the model has been calibrated to capture the dynamics of the population mean not the median).

mean but does not reduce variation stemming from differences between cells.

To further support this conclusion, and to be able to compare iCL and pCL more directly, we performed another experiment with four groups of 49 cells each where we switched the controller from pCL to iCL after 800 minutes (Supplementary Figure 16). Thereby, we calculated the population mean fluorescence during the iCL phase based on real-time classification of cells in the same way as during the pCL phase. Note, however, that in iCL control all cells remain independent and are not coupled through the mean as in pCL control. Hence, this classification only affects how the data is displayed and compared to the pCL data, but has no influence on the operation of the controller. Again, it can be observed that for all four cell groups pCL control lead to successful tracking of the mean but iCL control is needed to reduce cell-to-cell variability. The histograms displayed on the right of the panels show that during the pCL control phase the cells' fluorescence is widely, and almost uniformly, spread around the target profile. iCL control, on the other hand, leads to significantly more cells being close to the target and low variance between cells that only becomes comparable in size to the variance in the pCL phase when outlier cells that do not respond to control signals anymore are present.

Supplementary References

[1] P. Kirk, A. Babtie, and M. Stumpf. Systems biology (un)certainties. *Science*, 350 (6259):386–388, 2015.

[2] S. Engblom. Computing the moments of high dimensional solutions of the master equation. *Applied Mathematics and Computation*, 180(2):498–515, 2006.

[3] J. Ruess. Minimal moment equations for stochastic models of biochemical reaction networks with partially finite state space. *The Journal of Chemical Physics*, 143 (24):244103, 2015.

[4] E. Lakatos, A. Ale, P. Kirk, and M. Stumpf. Multivariate moment closure techniques for stochastic kinetic models. *The Journal of Chemical Physics*, 143(9):094107, 2015.

[5] C. Zechner, J. Ruess, P. Krenn, S. Pelet, M. Peter, J. Lygeros, and H. Koeppl. Moment-based inference predicts bimodality in transient gene expression. *Proceedings of the National Academy of Sciences of the USA*, 109(21):8340–8345, 2012. doi: 10.1073/pnas.1200161109.

[6] J. Ruess, A. Milias-Argeitis, and J. Lygeros. Designing experiments to understand the variability in biochemical reaction networks. *Journal of the Royal Society Interface*, 10(88):20130588, 2013. doi: 10.1098/rsif.2013.0588.

[7] J. Ruess and J. Lygeros. Moment-based methods for parameter inference and experiment design for stochastic biochemical reaction networks. *ACM Transactions on Modeling and Computer Simulation (TOMACS)*, 25(2):8, 2015.

[8] C. Zechner, M. Unger, S. Pelet, M. Peter, and H. Koeppl. Scalable inference of heterogeneous reaction kinetics from pooled single-cell recordings. *Nature Methods*, 11:197–202, 2015.

[9] L. Huang, L. Pauleve, C. Zechner, M. Unger, A. Hansen, and H. Koeppl. Reconstructing dynamic molecular states from single-cell time series. *Journal of the Royal Society Interface*, 13(122):20160533, 2016.

[10] A. Milias-Argeitis, M. Rullan, S. Aoki, P. Buchmann, and M. Khammash. Automated optogenetic feedback control for precise and robust regulation of gene expression and cell growth. *Nature Communications*, 7:12546, 2016.
