# Supplementary material for: Shaping bacterial population behavior through computer-interfaced control of individual cells
Source: Nat Commun. 2017 Nov 16;8:1535. doi: 10.1038/s41467-017-01683-1 (PMC5688142; doi:10.1038/s41467-017-01683-1)
Supplement: Supplementary file 2 — Description of Additional Supplementary Files [file 41467_2017_1683_MOESM2_ESM.pdf]

Description of Additional Supplementary Files

File Name: Supplementary Movie 1
Description: **Individually controlled mother cells with cell stimulation overlay**. Escherichia coli, expressing constitutive Venus YFP (grey channel) and optogenetically-regulated Cerulean CFP (cyan channel), grow outwards from the center of the fluorescence image, in horizontal channels of a microfluidic mother machine device. The registered control image projected onto the cells is overlaid in red and green (down- and up-regulating CFP, respectively). Mother cells' CFP expression is recorded and the cells are stimulated every six minutes at detection and stimulation regions (Supplementary Figure 2), respectively. CFP expression is suppressed and CFP diluted during a 600 minute pre-adaptation. CFP expression in each cell is then open loop (OL) controlled using precomputed light sequences, to one of two fluorescence targets (see Figure 3b). After 780 minutes, control is switched to individual closed loop (iCL), which adjusts stimulation sequences for the learned responsiveness of each cell. Cells and locations are continuously reviewed for invalidation due to pathological behavior (e.g., plasmid or mother cell loss, growth arrest, poor data quality. See Methods). Cells receive red/green stimulation signals even when invalidated.

File Name: Supplementary Movie 2
Description: **Sample cells tracking a target trajectory through 60 hours.** Individual closed loop (cell 1) and open loop (cell 2) controlled cerulean CFP expression (Upper image panels: cyan channel. Third panel: blue and orange lines) is shown for Escherichia coli tracking a variable expression target (red line, third panel). Outlines of the mother cell and daughters (yellow) are extracted from fluorescence images of constitutively-expressed venus YFP. Grey boxes in the image panels indicate mother cell data acquisition regions, and image extents represent the boundary of the light stimulation region. Red and green stimulation sequences for the cells are displayed at the bottom of the image panels. Controller-estimated cell responsiveness (unused in open loop control of cell 2) is displayed in panel 4.

File Name: Supplementary Movie 3
Description: **Response variation between two open-loop controlled cells.** Differing responses of two cells with identically open loop-controlled cerulean CFP expression (Upper image panels: cyan channel. Third panel: dark orange and orange lines) tracking a variable expression target (red line, third panel) over 30 hours. Outlines of the mother cell and daughters (yellow) are extracted from fluorescence images of constitutively-expressed venus YFP. Grey boxes in the image panels indicate mother cell data acquisition regions, and image extents represent the boundary of the light stimulation region. Red and green stimulation sequences for the cells are displayed at the bottom of the image panels. Controller-estimated cell responsiveness (unused in open loop control) is shown in panel 4. Cell 1 (fluorescence trajectory indicated with an asterisk in Figure 3a) undergoes large fluctuations in expression (and estimated responsiveness) relative to cell 2, which more closely tracks the mean open loop response in Figure 3a.

File Name: Supplementary Movie 4
Description: **Sample cells tracking two static expression targets under open loop and then individual closed loop control.** Cerulean CFP expression (Upper image panels: cyan channel. Third panel: purple and teal lines) tracking targets of 20 (cell 1) and 10 (cell 2) fluorescence units. Initial pre-computed open loop control switches to individual closed loop control after loop 230 (dashed vertical line, lower panels). Outlines of the mother cell and daughters (yellow) are extracted from fluorescence images of constitutively-expressed venus YFP. Grey boxes in the image panels indicate mother cell data acquisition regions, and image extents represent the boundary of the light stimulation region. Red and green stimulation sequences for the cells are displayed at the bottom of

the image panels. Controller-estimated cell responsiveness (unused in the open loop control interval) is shown in panel 4.

File Name: Supplementary Movie 5
Description: **Fluorescence trajectories of four, uncoupled, hybrid oscillators**. Four cells containing hybrid oscillator circuits (see Fig 5b,c), display unsynchronized oscillations in cerulean CFP fluorescence (lower panel). CFP fluorescence images in upper panels are of mother cell data acquisition regions.

File Name: Supplementary Movie 6
Description: **Fluorescence trajectories of four, positively-coupled, hybrid oscillators**. Four cells containing hybrid oscillator circuits (see Fig 5d,e), are positively coupled in a ring (10% of each cell's signal is transferred to each of its nearest neighbours, per time step). The cells display synchronized oscillations in cerulean CFP fluorescence (lower panel). CFP fluorescence images in upper panels are of mother cell data acquisition regions.

File Name: Supplementary Movie 7
Description: **Fluorescence trajectories of four, negatively-coupled, hybrid oscillators.** Four cells containing hybrid oscillator circuits (see Fig 5d,f), are negatively coupled in a ring (10% of each cell's signal is removed from each of its nearest neighbours, per time step). The cells converge to oscillations in cerulean CFP fluorescence with a half phase shift between neighbours (lower panel). CFP fluorescence images in upper panels are of mother cell data acquisition regions.

File Name: Supplementary Movie 8
Description: **Fluorescence trajectories of four, asymmetrically-coupled, hybrid oscillators**. Four cells containing hybrid oscillator circuits (see Fig 5d,g), are negatively coupled in one direction and positively-coupled in the other, along a ring (10% signal of each cell's signal is added or subtracted from its left and right nearest neighbours, respectively, per time step). The cells display cerulean CFP fluorescence oscillations with an approximately quarter phase shift between neighbours (lower panel). CFP fluorescence images in upper panels are of mother cell data acquisition regions.

File Name: Supplementary Software
Description: MATLAB scripts and supporting files for operating experimental hardware and performing automated closed loop feedback control of expression in single cells.
