# Supplementary material for: Shaping bacterial population behavior through computer-interfaced control of individual cells
Source: Nat Commun. 2017 Nov 16;8:1535. doi: 10.1038/s41467-017-01683-1 (PMC5688142; doi:10.1038/s41467-017-01683-1)
Supplement: Supplementary file 11 — Supplementary Software [file 41467_2017_1683_MOESM11_ESM.zip › readme.docx]

**Supplementary Software |** MATLAB scripts and supporting files for operating experimental hardware and performing automated closed loop feedback control of expression in single cells.

Note that this software is a work in progress that would welcome refinement. It may be used as a guideline, but is not plug and play. Portions will likely require adaptation to a particular physical experimental setup in order to function properly, and to avoid damage to the setup. Performance of each part and the whole should therefore be carefully verified when adapting it for use. The automated hardware components of the setup described in the manuscript, for which the software was developed, consist of:

- Olympus IX83 2-deck inverted microscope with UPLSAPO100XOPH objective
- Märzhäuser stage
- Hamamatsu Orca Flash4.0v2 SCMOS camera
- Lumencor SpectraX fluorescence illuminator
- Panasonic PT-AE6000E projector, configured as a second monitor
- TOFRA filter cube slider
- Arduino LED shutter controller

The physical setup was interfaced with a computer (Windows 7 (x64), 128GB RAM), installed with:

- MATLAB (version 2014b, with statistics, image processing, distributed computing toolboxes)
- MicroManager (v1.4.19) api (called in MATLAB for control of microscope and other hardware).
  - Note that for very fast cameras and stages, and oil objectives, a custom variant of the micromanager OughtaFocus function with a pause between acquisitions (source: OughtaFocusSleep.java) may be used to permit equilibration after z-axis steps.
- SUNDIALS (ODE solvers used in MATLAB for model-based controllers)
- mexOpenCV (used in MATLAB for mapping camera to projector images)

The following scripts, functions, and additional data are included:

- **holoManMultipoint.m [experiment manager script]**
  - **getIm.m [snap image]**
  - **holoAcq.m [image setup, acquisition, deshade]**
    - getIm.m
  - **holoAcqSetup.m [acquisition state configurator]**
  - **holoCon.m [send cell data to controllers, gather responses]**
  - **holoFoc.m [set focal offsets, focus]**
    - getIm.m
    - holoAcq.m
    - splash.m
    - **OughtaFocusSleep.java [autofocus w/ settling interval if needed, else use oughtaFocus]**
  - **holoFun.m [define image data to stimulus pipelines]**
    - holoCon.m
    - holoLoc.m
    - holoLog.m
    - **SundialsTB [ODE solvers]**
  - **holoLog.m [experiment log]**
  - **holoPro.m [stimulus projection]**
    - getIm.m
    - holoLog.m
    - proMicHomog.m
    - splash.m
  - **holoReg.m [image registration]**
  - **holoShd.m [load fluo shading correctors]**
  - **holoTrg.m [experiment-specific targets, light sequences, params]**
    - **SmileyBW24.png [bw smiley face for target]**
    - **happyTarg24Gen.m [smiley face target generator]**
    - **istTargGen.m [‘IST’ target generator]**
    - **lightSeq160318.mat [test light sequence]**
    - **lightSeq160322.mat [test light sequence]**
    - **lightSeq160601.mat [test light sequence]**
  - **proMicHomog.m [image perspective transform]**
    - **mexOpenCV [image maps]**
  - **proMicHomogGet.m [projector-camera image map]**
    - getIm.m
    - proMicHomog.m
    - splash.m
    - mexOpenCV
  - **holoLoc.m [image location definition, data, and stimuli]**
    - holoLog.m
  - **splash.m [project image]**
- **holoVld.m [test cell validity]**
- **imShd_CFP.mat [CFP shading image]**
- **imShd_YFP.mat [YFP shading image]**

Controllers:

- **holoCon_00_nullController.m**

**[no effect, placeholder]**

- **holoCon_co_nonlinearFreeHiddenDyn_str138.m**

**[model-predictive closed loop, large h2, R=10]**

- **holoCon_co_nonlinearFreeHiddenDyn_str138_R1.m**

**[model-predictive closed loop, large h2, R=1]**

- **holoCon_co_nonlinearFreeHiddenDyn_str138_R100.m**

**[model-predictive closed loop, large h2, R=100]**

- **holoCon_co_nonlinearSlowHiddenDyn_str138.m**

**[model-predictive closed loop, small h2, R=10]**

- **holoCon_co_nonlinearSlowHiddenDyn_str138_R1.m**

**[model-predictive closed loop, small h2, R=1]**

- **holoCon_co_nonlinearSlowHiddenDyn_str138_R100.m**

**[model-predictive closed loop, small h2, R=100]**

- **holoCon_co_nonlinearFreeHiddenDyn_str138_muZ2.m**

**[model-predictive closed loop, standard: small h2, R=10, muZ=2]**

- **holoCon_oc_nonlinearFreeHiddenDyn_str138_muZ2_c130o400.m**

**[model-predictive, 130 cycle closed-400 cycle open loop, standard]**

- **holoCon_oc_nonlinearFreeHiddenDyn_str138_muZ2.m**

**[model-predictive open loop, standard: small h2, R=10, muZ=2]**

- **holoCon_oc_nonlinearFreeHiddenDyn_str138_muZ2_o130c400.m**

**[model-predictive, 130 cycle open-400 cycle closed loop, standard]**

- **holoCon_oc_nonlinearFreeHiddenDyn_str138_muZ2_pCL130iCL400.m**

**[model-predictive, 130 cycle pCL-400 cycle iCL, standard]**

- **holoCon_oc_nonlinearFreeHiddenDyn_str138_muZ2_pCL130iCL400_rbst.m**

**[model-predictive, 130 cycle pCL-400 cycle iCL, standard, w/cell validation]**

- **holoCon_oc_nonlinearFreeHiddenDyn_str138_muZ2_pCL600iCL400.m**

**[model-predictive, 600 cycle pCL-400 cycle iCL, standard]**

- **holoCon_oc_nonlinearFreeHiddenDyn_str138_muZ2_pCL600iCL400_rbst.m**

**[model-predictive, 600 cycle pCL-400 cycle iCL, standard, w/cell validation]**

- **holoCon_tx_swingers.m**

**[hybrid oscillators]**

In general, once equipment-specific parameters are determined (e.g., control and data channels, exposure times, projector screen position,…), experiments are specified by: (1) associating cells with selected controller functions (holoCon_xx_yyyyyy.m), and (2) setting ‘targetN’ in holoManMultipoint.m, to configure parameters by holoTrg.m such as function ordering (‘funN’), control targets, and open loop light sequences. For example, we conducted the experiments for Figures 3-5 with the following settings:

| **Figure** | **targetN** | **controller(s)** |
| --- | --- | --- |
| Figure 3a | 1.626080 | holoCon_co_nonlinearFreeHiddenDyn_str138_muZ2.m  holoCon_oc_nonlinearFreeHiddenDyn_str138_muZ2.m |
| Figure 3b | 3.36 | holoCon_oc_nonlinearFreeHiddenDyn_str138_muZ2_o130c400.m |
| Figure 3c | 4.24 | holoCon_co_nonlinearFreeHiddenDyn_str138_muZ2 |
| Figure 4 | 3.26 | holoCon_co_nonlinearFreeHiddenDyn_str138_muZ2.m  holoCon_oc_nonlinearFreeHiddenDyn_str138_muZ2.m |
| Figure 5 | 10.1 | holoCon_tx_swingers.m |
