# Supplementary material for: Shaping bacterial population behavior through computer-interfaced control of individual cells
Source: Nat Commun. 2017 Nov 16;8:1535. doi: 10.1038/s41467-017-01683-1 (PMC5688142; doi:10.1038/s41467-017-01683-1)
Supplement: Supplementary file 11 — Supplementary Software [file 41467_2017_1683_MOESM11_ESM.zip › splash.m license.docx]

The function splash.m in this folder is derivative of splash.m (source below), obtained from [MathWorks File Exchange](https://de.mathworks.com/matlabcentral/fileexchange/5946-splash-m-revised-) with the following license:

Copyright (c) 2009, Qun HAN 
All rights reserved.

Redistribution and use in source and binary forms, with or without 
modification, are permitted provided that the following conditions are 
met:

* Redistributions of source code must retain the above copyright 
notice, this list of conditions and the following disclaimer. 
* Redistributions in binary form must reproduce the above copyright 
notice, this list of conditions and the following disclaimer in 
the documentation and/or other materials provided with the distribution

THIS SOFTWARE IS PROVIDED BY THE COPYRIGHT HOLDERS AND CONTRIBUTORS "AS IS" 
AND ANY EXPRESS OR IMPLIED WARRANTIES, INCLUDING, BUT NOT LIMITED TO, THE 
IMPLIED WARRANTIES OF MERCHANTABILITY AND FITNESS FOR A PARTICULAR PURPOSE 
ARE DISCLAIMED. IN NO EVENT SHALL THE COPYRIGHT OWNER OR CONTRIBUTORS BE 
LIABLE FOR ANY DIRECT, INDIRECT, INCIDENTAL, SPECIAL, EXEMPLARY, OR 
CONSEQUENTIAL DAMAGES (INCLUDING, BUT NOT LIMITED TO, PROCUREMENT OF 
SUBSTITUTE GOODS OR SERVICES; LOSS OF USE, DATA, OR PROFITS; OR BUSINESS 
INTERRUPTION) HOWEVER CAUSED AND ON ANY THEORY OF LIABILITY, WHETHER IN 
CONTRACT, STRICT LIABILITY, OR TORT (INCLUDING NEGLIGENCE OR OTHERWISE) 
ARISING IN ANY WAY OUT OF THE USE OF THIS SOFTWARE, EVEN IF ADVISED OF THE 
POSSIBILITY OF SUCH DAMAGE.

Original source code:

function varargout = splash(varargin)

%SPLASH Creates a splash screen.

% SPLASH(FILENAME,FMT,TIME) creates a splash screen using the image from the

% file specified by the string FILENAME, where the string FMT specifies

% the format of the file and TIME is the duration time of the splash

% screen in millisecond. If the file is not in the current directory or in a

% directory in the MATLAB path,specify the full pathname of the location

% on your system. If SPLASH cannot find a file named FILENAME, it looks

% for a file named FILENAME.FMT.

%

% SPLASH(FILNAME,FMT) creates a splash screen with the default duration time(3s)

%

% HSPLASH = SPLASH(FILENAME,FMT) creates a splash screen and returns its handle

% in HSPLASH. The splash screen will be always shown until SPLASH(HSPLASH,'off')

% is called to turn it off.

%

% SPLASH(HSPLASH,'off') closes the splash screen with handle HSPLASH. Since

% HSPLASH is actually a javax.swing.JWindow object, you can also close it

% by HSPLASH.dispose();

%

% Supported file types ---- formats supported by the imread function

% --------------------

% JPEG Any baseline JPEG image; JPEG images with some

% commonly used extensions; 8-bit and 12-bit lossy

% compressed RGB and grayscale images; 8-bit and 12-bit

% lossless compressed RGB images; 8-bit, 12-bit, and

% 16-bit lossless compressed grayscale images

%

% TIFF Any baseline TIFF image, including 1-bit, 8-bit, and

% 24-bit uncompressed images; 1-bit, 8-bit, and 24-bit

% images with packbits compression; 1-bit images with

% CCITT compression; 16-bit grayscale, 16-bit indexed,

% and 48-bit RGB images; 24-bit and 48-bit ICCLAB

% and CIELAB images; 32-bit and 64-bit CMYK images; and

% 8-bit tiled TIFF images with any compression and colorspace

% combination listed above.

%

% GIF Any 1-bit to 8-bit GIF image

%

% BMP 1-bit, 4-bit, 8-bit, 16-bit, 24-bit, and 32-bit uncompressed

% images; 4-bit and 8-bit run-length encoded (RLE) images

%

% PNG Any PNG image, including 1-bit, 2-bit, 4-bit, 8-bit,

% and 16-bit grayscale images; 8-bit and 16-bit

% indexed images; 24-bit and 48-bit RGB images

%

% HDF 8-bit raster image datasets, with or without an

% associated colormap; 24-bit raster image datasets

%

% PCX 1-bit, 8-bit, and 24-bit images

%

% XWD 1-bit and 8-bit ZPixmaps; XYBitmaps; 1-bit XYPixmaps

%

% ICO 1-bit, 4-bit, and 8-bit uncompressed images

%

% CUR 1-bit, 4-bit, and 8-bit uncompressed images

%

% RAS Any RAS image, including 1-bit bitmap, 8-bit indexed,

% 24-bit truecolor and 32-bit truecolor with alpha.

%

% PBM Any 1-bit PBM image. Raw (binary) or ASCII (plain) encoded.

%

% PGM Any standard PGM image. ASCII (plain) encoded with

% arbitrary color depth. Raw (binary) encoded with up

% to 16 bits per gray value.

%

% PPM Any standard PPM image. ASCII (plain) encoded with

% arbitrary color depth. Raw (binary) encoded with up

% to 16 bits per color component.

%

% Example(1)

% % To show 'splash.png' on a splash screen for 3 seconds you can call one of the

% % following three commands:

% splash('splash','png',3000);

% splash('splash.png',3000);

% splash('splash.png');

% Example(2)

% % To show a splash screen while you GUI program is initialized

% h = splash('splash','png');

% ............;% Place you GUI initializing code here

% splash(h,'off'); % Close the splash screen

% ............; % Other commands

%

% Example(3)

% % Use splash screen with GUIDE generated files

% h = splash('splash','png');% as the FIST line of the GUI opening function

% ........................;% other opening code

%

% splash(h,'off'); % as the last line of the GUI opening function

% Note

% The splash screen is created through the JAVA interface of MATLAB.

% Java requires uint8 data to create an instance of the Java image class,

% java.awt.Image. If the input image is of class uint8, jimage contains

% the same uint8 data. If the input image is of class double or uint16,

% im2java makes an equivalent image of class uint8, rescaling or offsetting

% the data as necessary, and then converts this uint8 representation to an

% instance of the Java image class, java.awt.Image. So some image formats may

% appear different form the source image in the splash screen created by SPLASH.

% To reduce the distortion, the following image formats are recommend: JPEG,PNG,

% BMP,TIFF,PCX,ICO.

%

% See also imread,im2java

% Han Qun, Sept. 2004

% Copyright 2004-2005 Han Qun

% College of Precision Instrument and Opto-Electronics Engineering,

% Tianjin University, 300072, P.R.China.

% Email: junziyang@126.com

% $Revision: 1.2 $ $Date: 2005/12/2 17:16:48 $

%% Check output

if nargout >=2

error('MATLAB:splash','%s','Too many output!');

end

%% Check input

[filename, format, time, handle, msg] = parse_inputs(varargin{:});

if (~isempty(msg))

error('MATLAB:splash:inputParsing', '%s', msg);

end

if (~isempty(handle))

handle.dispose;

return;

end

%% Load image

try

fullName = filename;

if ~isempty(format)

fullName = strcat(filename,'.',format);

end

I = imread(fullName);

catch

err = lasterror;

error('MATLAB:splash:imread','%s',err.message);

end

%% Create splash screen

splashImage = im2java(I);

win = javax.swing.JWindow;

icon = javax.swing.ImageIcon(splashImage);

label = javax.swing.JLabel(icon);

win.getContentPane.add(label);

win.setAlwaysOnTop(true);

win.pack;

%% set the splash image to the center of the screen

screenSize = win.getToolkit.getScreenSize;

screenHeight = screenSize.height;

screenWidth = screenSize.width;

% get the actual splashImage size

imgHeight = icon.getIconHeight;

imgWidth = icon.getIconWidth;

win.setLocation((screenWidth-imgWidth)/2,(screenHeight-imgHeight)/2);

win.show % show the splash screen

%% Output the handle

if (nargout==1)

varargout{1} = win;

time = [];

wn = 'Input duration time is discarded. Use SPLASH(handle,''off'') to close it';

warning('MTLAB:splash','%s',wn);

end

%% controling the duration time

if ~isempty(time)

tic;

while toc < time/1000

end

win.dispose() % close the splash screen

end

%% Function parse_inputs

function [filename, format, time, handle, msg] = parse_inputs(varargin)

filename = '';

format = '';

time = 3000; % Default 3 seconds

handle = [];

msg = '';

% Parse arguments based on their number.

switch(nargin)

case 0 % Not allowed.

msg = 'Too few input arguments.';

return;

case 1 % Filename only.

filename = varargin{1};

case 2 % Filename+format or filename+ time or handle+'off'

in1 = varargin{1};

in2 = varargin{2};

if ischar(in1) && ischar(in2) % Filename and format

filename = in1;

format = in2;

elseif ischar(in1) && isnumeric(in2)

filename = in1;

time = in2;

elseif isjava(in1) && isequal(in2,'off')

handle = in1;

else

msg='Input type mismatch. Help splash for more information';

end

case 3

in1 = varargin{1};

in2 = varargin{2};

in3 = varargin{3};

if ischar(in1) && ischar(in2) && isnumeric(in3)

filename = in1;

format = in2;

time = in3;

else

msg='Input type mismatch. Help splash for more information';

end

otherwise

msg = 'Too many input arguments.';

end
