# Supplementary figures and images for: Shaping bacterial population behavior through computer-interfaced control of individual cells
Source: Nat Commun. 2017 Nov 16;8:1535. doi: 10.1038/s41467-017-01683-1 (PMC5688142; doi:10.1038/s41467-017-01683-1)

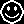

Supplement: Supplementary file 11 — Supplementary Software [file 41467_2017_1683_MOESM11_ESM.zip › SmileyBW24.png]
